# Disentangling associations between multiple environmental exposures and all-cause mortality: an analysis of European administrative and traditional cohorts

**DOI:** 10.3389/fepid.2023.1328188

**Published:** 2024-01-12

**Authors:** Konstantina Dimakopoulou, Federica Nobile, Jeroen de Bont, Kathrin Wolf, Danielle Vienneau, Dorina Ibi, Fabián Coloma, Regina Pickford, Christofer Åström, Johan Nilsson Sommar, Maria-Iosifina Kasdagli, Kyriakos Souliotis, Anastasios Tsolakidis, Cathryn Tonne, Erik Melén, Petter Ljungman, Kees de Hoogh, Roel C. H. Vermeulen, Jelle J. Vlaanderen, Klea Katsouyanni, Massimo Stafoggia, Evangelia Samoli

**Affiliations:** ^1^ Department of Hygiene, Epidemiology and Medical Statistics, Medical School, National and Kapodistrian University of Athens, Athens, Greece; ^2^ Department of Epidemiology, Lazio Region Health Service/ASL Roma 1, Rome, Italy; ^3^ Institute of Environmental Medicine, Karolinska Institutet, Stockholm, Sweden; ^4^ Institute of Epidemiology, Helmholtz Zentrum München, German Research Center for Environmental Health, Neuherberg, Germany; ^5^ Department of Epidemiology and Public Health, Swiss Tropical and Public Health Institute, Basel, Switzerland; ^6^ University of Basel, Basel, Switzerland; ^7^ Institute for Risk Assessment Sciences (IRAS), Utrecht University, Utrecht, Netherlands; ^8^ Barcelona Institute for Global Health (ISGlobal), Barcelona, Spain; ^9^ Department of Public Health and Clinical Medicine, Umeå University, Umeå, Sweden; ^10^ Department of Social and Education Policy, University of Peloponnese, Corinth, Greece; ^11^ Health Policy Institute, Athens, Greece; ^12^ IDIKA SA—e-Government Center for Social Security Services, Athens, Greece; ^13^ Universitat Pompeu Fabra (UPF), Barcelona, Spain; ^14^ CIBER Epidemiología y Salud Pública (CIBERESP), Madrid, Spain; ^15^ Department of Clinical Sciences and Education, Södersjukhuset, Karolinska Institutet, Stockholm, Sweden; ^16^ Sachś Children and Youth Hospital, Södersjukhuset, Stockholm, Sweden; ^17^ Department of Cardiology, Danderyd Hospital, Stockholm, Sweden; ^18^ MRC Centre for Environment and Health, Environmental Research Group, Imperial College London, United Kingdom NIHR HPRU in Environmental Exposures and Health, Imperial College London, London, United Kingdom

**Keywords:** administrative cohorts, traditional adult cohorts, all-cause mortality, external exposome, air pollution, NDVI, ambient temperature

## Abstract

**Background:**

We evaluated the independent and joint effects of air pollution, land/built environment characteristics, and ambient temperature on all-cause mortality as part of the EXPANSE project.

**Methods:**

We collected data from six administrative cohorts covering Catalonia, Greece, the Netherlands, Rome, Sweden, and Switzerland and three traditional cohorts in Sweden, the Netherlands, and Germany. Participants were linked to spatial exposure estimates derived from hybrid land use regression models and satellite data for: air pollution [fine particulate matter (PM_2.5_), nitrogen dioxide (NO₂), black carbon (BC), warm season ozone (O_3_)], land/built environment [normalized difference vegetation index (NDVI), distance to water, impervious surfaces], and ambient temperature (the mean and standard deviation of warm and cool season temperature). We applied Cox proportional hazard models accounting for several cohort-specific individual and area-level variables. We evaluated the associations through single and multiexposure models, and interactions between exposures. The joint effects were estimated using the cumulative risk index (CRI). Cohort-specific hazard ratios (HR) were combined using random-effects meta-analyses.

**Results:**

We observed over 3.1 million deaths out of approximately 204 million person-years. In administrative cohorts, increased exposure to PM_2.5_, NO_2_, and BC was significantly associated with all-cause mortality (pooled HRs: 1.054, 1.033, and 1.032, respectively). We observed an adverse effect of increased impervious surface and mean season-specific temperature, and a protective effect of increased O_3_, NDVI, distance to water, and temperature variation on all-cause mortality. The effects of PM_2.5_ were higher in areas with lower (10th percentile) compared to higher (90th percentile) NDVI levels [pooled HRs: 1.054 (95% confidence interval (CI) 1.030–1.079) vs. 1.038 (95% CI 0.964–1.118)]. A similar pattern was observed for NO_2_. The CRI of air pollutants (PM_2.5_ or NO_2_) plus NDVI and mean warm season temperature resulted in a stronger effect compared to single-exposure HRs: [PM_2.5_ pooled HR: 1.061 (95% CI 1.021–1.102); NO_2_ pooled HR: 1.041 (95% CI 1.025–1.057)]. Non-significant effects of similar patterns were observed in traditional cohorts.

**Discussion:**

The findings of our study not only support the independent effects of long-term exposure to air pollution and greenness, but also highlight the increased effect when interplaying with other environmental exposures.

## 1 Introduction

Epidemiological studies have documented the adverse health effects of long-term exposure to air pollution, particularly of particulate matter with a diameter under 2.5 µm (PM_2.5_), which ranks as the fifth risk factor for death worldwide according to the Global Burden of Disease Study (1). The recent revision of the air quality guidelines by the World Health Organization (WHO) was informed by Chen and Hoek (2), which concluded that exposure to PM_2.5_ is associated with increased mortality from natural, cardiovascular, and respiratory causes, as well as by Huangfu and Atkinson (3), who reported positive associations between long-term exposure to nitrogen dioxide (NO_2_) and natural mortality. Fewer studies have evaluated the association of long-term exposure to ozone (O_3_) with mortality (3, 4) with inconsistent results. As an emerging pollutant of interest, black carbon (BC) has been associated with effects on natural mortality, with heterogeneity between study-specific estimates (5).

Ambient temperature is another ubiquitous environmental exposure that has been associated with adverse health effects after short-term exposure (6–9). Few studies have assessed the associations of long-term temperature exposure and health (10), indicating potential associations apart from a short-term period, especially with cardiovascular outcomes.

A growing body of literature has indicated the beneficial health effects of living near natural environments (11, 12) linked with stress reduction, better mental health and wellbeing, promotion of physical activity, social interactions, and a potential reduction of air pollution and noise levels (11, 13–19). Recent meta-analyses support a protective association between exposure to greenness and natural-cause mortality (12, 20), while the association with proximity to blue spaces has provided inconsistent results so far (21–25).

Previous findings have overwhelmingly focused on single specific exposures, such as air pollution, temperature, and green space (26), while the complex interactions accounting for the simultaneous human exposure to all these agents remain less studied (27), partly due to several constraints, including the exposure assessment and the methodological approaches and their related interpretation. Studying the single-exposure effects is a straightforward procedure, but, in fact, populations are exposed to a number of environmental stressors simultaneously. Therefore, the investigation of the combined effect of multiexposures on human health is of growing interest. The external exposome framework attempts to address the totality of environmental exposures experienced over the life course (28), including characteristics of the physical environment, such as air pollution, the land/built environment, green, gray, and blue spaces, and meteorological conditions, including temperature indices.

Within the EXposome Powered tools for healthy living in urbAN Settings (EXPANSE) (26) project, we applied a common exposure assessment and epidemiological analysis. The objective of this study was to disentangle the complex associations between multiple *a priori* selected environmental exposures and all-cause mortality in six administrative cohorts and three traditional adult cohorts spanning across Europe. The different cohort designs complement potential findings and may inform sources of heterogeneity for the effect estimates attributed to the design and residual confounding.

## 2 Methods

### 2.1 Study population and outcome

We collected data from six administrative cohorts and three traditional prospective cohorts. More specifically, the administrative cohorts were four country-wide cohorts, namely in Greece, the Netherlands, Sweden, and Switzerland, one region-wide cohort for Catalonia, Spain and one city-wide cohort in Rome, Italy (29–32). The traditional cohorts were: the Cardiovascular Effects of Air Pollution and Noise in Stockholm (CEANS) study in Sweden; the European Prospective Investigation into Cancer and Nutrition in the Netherlands (EPIC-NL) in the Netherlands; and the Cooperative Health Research in the Region of Augsburg (KORA) in Germany (33–35).

The administrative cohorts included all adults aged 37 years and above. The baseline period ranged between 2010 and 2014, and the end of the study period was either 2018 or 2019. In the traditional cohorts, participants were recruited between 1992 and 2004 and the end of the study period was between 2011 and 2015. The total sample size exceeded 27.5 million participants. All participants were followed up until death, migration, loss of follow-up, or the end of the study period, whichever occurred first. Participants from the administrative cohorts aged under 37 years and participants whose residential address was missing were excluded from the analysis. The requirement for participants to be aged 37 years and older was chosen to match the availability of data in the Greek administrative cohort for consistency across cohorts. Detailed cohort-specific descriptions are provided in the Supplementary Material (Appendix I). Cohort-specific analyses were conducted locally in each center using a common protocol and R code, while the data were extracted and recorded according to a shared codebook. The original cohort studies were approved by corresponding institutional review boards, complying with all relevant national, state, and local regulations. The EXPANSE project was conducted in accordance with the Declaration of Helsinki. The outcome was all-cause mortality and was provided through linkage with mortality registries (International Classification of Diseases codes: ICD-9: 001–799, ICD-10: A00–R99). Note that all-cause mortality was chosen since it has the least potential of misclassification compared to cause-specific mortality and therefore is the most “stable” outcome in terms of interpreting the study findings. In both cohort types, participants were followed up until death from all causes, emigration out of the study area, or the end of the study, whichever came first.

### 2.2 Exposure assessment

We used a harmonized exposure assessment protocol developed within the EXPANSE project (26) to assign predicted exposures to the geocoded residential addresses of the participants in each cohorts. Selected environmental exposures were grouped *a priori* in three exposure “domains”: (A) ambient air pollution; (B) land/built environment; and (C) temperature (Supplementary Material Appendix II, Table S1).

#### 2.2.1 Ambient air pollution domain

We used the predictions of the annual average concentrations for PM_2.5_, NO_2_, BC, and O_3_ during the warm season (April to September) derived from European-wide land use regression (LUR) models (36) for 2010 at a spatial resolution of 100 × 100 m. Supervised linear regression models were developed incorporating satellite data, chemical transport model estimates, road network, land use, and measurement data from routine monitoring stations. The models for PM_2.5_, NO_2_, BC, and O_3_ explained 66%, 58%, 51%, and 60% of the spatial variability of the measured concentrations, respectively.

#### 2.2.2 Built environment domain: greenness, gray, and blue spaces

We used the normalized difference vegetation index [NDVI (37)] as an indicator of greenness, which is measured on a scale of −1 to 1, with numbers closer to 1 indicating higher greenness (38). The index is based on the reflection of visible and near-infrared light by vegetation, while it is calculated by the difference between near-infrared and visible radiation divided by the sum of near-infrared and visible radiation (38). NDVI data were retrieved from the Vegetation Indices (MOD13Q1) product of the Terra Moderate Resolution Imaging Spectroradiometer (MODIS) with a spatial resolution of 250 × 250 m in 2019. We retrieved high-resolution data, measured as the percentage (%) of impervious surfaces, at 100 × 100 m for impervious density in 2015 from the Copernicus Land Monitoring Service (2020), representing the percentage of soil sealing per area unit used to assess gray spaces. The exposure to blue spaces was evaluated from the EU-Hydro baseline map (2011–2013) developed by the CLMS (Copernicus Land Monitoring Service, 2019) which provides high-resolution data on the river network, water bodies (e.g., lakes and wide rivers), drainage network with catchment areas, drainage lines, and sea/ocean water. We estimated the Euclidean distance from the residential address to the nearest blue space (39).

#### 2.2.3 Ambient temperature domain

We used the European Centre for Medium-Range Weather Forecasts (ECMWF) ERA5-Land reanalysis dataset for 2010 to assess the daily land surface temperature data (in °C) at a resolution of 11 × 11 km in Europe. The estimates were linked to each participant's residential address and then aggregated to calculate season-specific mean and standard deviation values (cool season: December to March; warm season: April to September).

### 2.3 Statistical analysis

The analysis for both cohort types followed a two-stage approach. In the first stage, each cohort applied Cox proportional hazard models, accounting for several cohort-specific individual and area-level variables. Age was used as time scale to better account for potential confounding by age (40). We applied three Cox models with successively more detailed control for individual- and area-level confounders (4). Individual-level information was limited in the administrative cohorts, hence the selection of the individual confounders differed between the traditional adult and administrative cohorts. For country-wide administrative cohorts, model 1 included age (timescale, in years), sex (as strata variables), and a dummy variable for the nomenclature of territorial units for statistics (NUTS-1). Model 2 included all individual-level variables available within each administrative cohort (specific adjusting variables are specified in the Results section as these depended on cohort). Model 3, in addition, included area-level variables mainly characterizing socioeconomic status (SES). For traditional adult cohorts, model 1 included age (timescale, in years), sex and subcohort, if applicable (both as strata variables), and the baseline year. Model 2 was further adjusted for individual lifestyle and socioeconomic information at baseline: marital status (single, married or living with partner, divorced or separated, or widowed); body mass index (BMI); smoking status (never, former, or current) and smoking duration (years); intensity of smoking among current and former smokers (cigarettes per day) and smoking intensity squared; employment status (yes or no); and level of education (primary or less, secondary, or tertiary, as per country-specific definitions). Model 3 also took into account area-level variables to characterize SES. Model 3 was considered our main model. Model 3 adjusts for the largest available per case number of potential confounders, including both individual- and area-level variables. The inclusion of various area-level covariates reflecting SES has been shown to compensate for the lack of individual lifestyle covariates in the administrative cohorts (41, 42). The spatial scale varied from square blocks (in cities in Greece with a population over 100,000) or small neighborhoods (CEANS, EPIC-NL) to municipalities (KORA and administrative cohorts with varying degree of spatial coverage). More details on the cohorts and covariate availability are given in the Supplementary Material (Appendix III). Participants with missing information in model 3 covariates were excluded from the analyses.

We evaluated the interplay between exposures by applying: (1) single-exposure models; and (2) adding an interaction term between selected exposures based on the *a priori* hypothesis that increased surroundings of green spaces may decrease air pollution or increased temperature effects. The hazard ratios (HR) and corresponding 95% confidence intervals (CI) for the interaction between air pollutants (PM_2.5_ or NO_2_), or mean temperature during the warm season and NDVI, were calculated as an increment per 5 µg/m^3^ in PM_2.5_, 10 µg/m^3^ in NO_2_, or 1°C in mean warm temperature at the 10th percentile of NDVI distribution (to assess the effect when exposed to low greenness) compared to the same increment in air pollutants and temperature exposure and the 90th percentile of NDVI distribution (to assess the effect when exposed to high greenness); (3) multiexposure models selecting one exposure of each exposure domain based on the minimization of the Bayesian Information Criterion (BIC); (4) the product of the HRs based on the survival models, including multiexposures, was interpreted as the cumulative risk index (CRI) and was used to estimate the joint risk of the exposures, by the same and across domains. The estimated CRI is the additive effects of joint exposures on all-cause mortality. Thus, the CRI represents the relative hazard for fixed-unit change in multiexposures compared with that for no change in any of the exposures. More details on the estimation of CRI can be found in the Supplementary Material (Appendix IV). Note that the CRI for NDVI was assessed per 0.1 unit decrease of NDVI to reflect a deterioration of the external exposome. Correlations of exposures were also considered. To avoid multicollinearity problems in the multiexposure models, we included only one exposure from each of the three exposure domains (air pollution, land/built environment, ambient temperature). Variance inflation factors (VIFs) were calculated as a measure of multicollinearity for all the covariates included in the final models.

Cohort-specific effect estimates obtained from the first stage were meta-analyzed under a random effects model using the Hartung–Knapp correction (for the small number of studies) and a restricted maximum likelihood estimator for between-studies variance. The effect estimates were pooled separately by the administrative and traditional adult cohort designs. All pooled HRs are based on the main model 3 results. Fixed exposure increments were used to calculate meta-analysis HRs and the corresponding 95% CIs: 10 µg/m^3^ for NO_2_; 5 µg/m^3^ for PM_2.5_; 0.5 (10^−5^/m) for BC; 10 µg/m^3^ for O_3_; 0.1 units for NDVI; 10% units for impervious surfaces; 1,000 m for distance to water; 1.0°C for mean temperature warm/cool and for SD temperature warm/cool season. We used the *I*^2^ statistics and Cochran's *Q* to quantify the heterogeneity among studies. All statistical analyses were conducted using R version 4.1.3.

## 3 Results

Table 1 summarizes the cohort characteristics by design: administrative and traditional adult cohorts. The total number of participants, after excluding individuals with missing values for any of the individual- or area-level variables, was 27,731,158 and 57,653 in the administrative and traditional adult cohorts, respectively; with 22% and 28.5% contributed by Greece and the Netherlands in the administrative cohorts, respectively, and 51% contributed by EPIC-Netherlands in the traditional adult cohorts. There were 3,132,704 (11.3%) total deaths from all causes in the administrative cohorts, ranging from 5% (Catalonia) to 14.5% (Sweden). The total number of deaths in the traditional adult cohorts was 5,605 (9.7%), ranging from 3.1% (EPIC-NL) to 19.9% (KORA). The mean age of the participants ranged from 49.8 years (KORA) to 60.3 years (Greece). The sex distribution ranged from 48.4% women (Sweden) to 77.1% women (EPIC-NL). More details on the cohorts and covariate availability are given in the Supplementary Material (Appendix III).

**Table 1:** Description of the administrative and traditional adult cohorts^a^ and participants’ characteristics.

Cohort	*N* total	*N* total deaths	Age (SD, in years)	Female (%)	Follow-up time (person-years)	All-cause mortality rate per 1,000 person-years	Study area
A. Administrative							
Catalonia	3,660,594	182,788	56.7 (14.1)	52.8	2015–2019 (17,475,621)	10.5	Catalonia, Spain
Greece	6,121,421	632,439	60.3 (15.3)	52.5	2014–2019 (29,141,748)	21.7	Greece, country-wide
Netherlands	7,909,565	1,064,637	53.3 (13.2)	50.6	2010–2019 (73,709,135)	14.4	Netherlands, country-wide
Rome	1,539,784	201,093	59.3 (14.5)	55.3	2011–2019 (11,463,921)	17.5	Rome, metropolitan area, Italy
Sweden	4,274,326	618,983	59.3 (13.9)	48.4	2010–2018 (35,835,230)	17.3	Sweden, country-wide
Switzerland	4,225,468	432,764	56.8 (13.4)	51.7	2010–2018 (35,648,810)	12.1	Switzerland, country wide
B. Traditional adult							
CEANS	19,888	3,036	56.4 (11.4)	57.8	1992–2011 (259,019)	11.7	Stockholm county, Sweden
EPIC-NL	29,428	912	50.6 (11.2)	77.1	1993–2016 (619,572)	1.5	Netherlands
KORA	8,337	1,657	49.8 (13.9)	50.5	1994–2001/2011–2014 (140,583)	11.8	Augsburg city + 2 adjacent counties, Germany

CEANS, cardiovascular effects of air pollution and noise in Stockholm study; EPIC-NL, the European prospective investigation into cancer and nutrition—Netherlands; KORA, cooperative health research in the Augsburg region.

^a^ Final sample size per cohort without any missing value included in main model 3.

The levels of air pollution, indicators of the land/built environment, and ambient temperature characterizing the participants' long-term exposure at their residences are shown in Table 2. A general geographical trend is observed, with lower air pollutant concentrations, higher NDVI, and lower mean annual temperature in the northern countries compared to the southern countries. Different cohort studies in the same country (e.g., CEANS and the administrative cohort in Sweden) displayed similar values. Most of the correlations were moderate, ranging from ±0.5 to ±0.7. The correlation coefficient *r* for PM_2.5_ and NDVI were in the range of −0.37 to −0.59, while for NO_2_ and NDVI, the range was −0.49 to −0.79. More information on the exposure assessment and the correlations between all exposures in each cohort are given in the Supplementary Material (Appendix II, IV, Tables S1,S2).

**Table 2:** Levels (median and interquartile range) of external exposome parameters by cohort design.

		A. Administrative cohorts						B. Traditional adult cohorts		
Domain	Exposure	Catalonia	Greece	Netherlands	Rome	Sweden	Switzerland	CEANS	EPIC-NL	KORA
Air pollutants	PM_2.5_ (µg/m^3^)	16.3 (2.5)	17.7 (3.1)	17.3 (2.1)	16.6 (1.1)	7.8 (2.2)	16.2 (2.6)	8.2 (1.0)	17.3 (1.4)	16.7 (0.9)
	NO_2_ (µg/m^3^)	36.7 (20.4)	22.9 (23.7)	31.3 (8.7)	32.7 (7.8)	14.3 (6.0)	22.6 (8.8)	19.2 (8.2)	34.3 (8.0)	23.0 (6.6)
	BC (10^−5^/m)	2.1 (0.7)	2.1 (0.9)	1.5 (0.4)	2.2 (0.4)	0.7 (0.4)	1.6 (0.4)	0.8 (0.4)	1.6 (0.4)	1.6 (0.4)
	O_3_ (µg/m^3^)	70.2 (11.6)	104.0 (14.8)	76.0 (5.7)	95.1 (3.6)	76.9 (1.3)	94.2 (7.1)	76.9 (2.7)	74.4 (5.7)	85.4 (2.9)
Land/Built environment	NDVI	0.2 (0.1)	0.3 (0.2)	0.5 (0.1)	0.4 (0.2)	0.5 (0.1)	0.6 (0.2)	0.5 (0.1)	0.5 (0.1)	0.5 (0.1)
	Impervious surface (%)	72.0 (29.0)	63.0 (40.0)	57.0 (35.0)	69.0 (32.0)	10.0 (31.0)	41.0 (33.0)	27.0 (34.0)	54.0 (36.0)	49.0 (26.0)
	Distance water (m)	1,500.0 (2,061.5)	3,087.1 (5,764.5)	1,000 (1,145.4)	1,897.4 (2,505.3)	1,300 (1,042.2)	1,272.8 (2,086.4)	700.0 (1,089.2)	948.7 (1,000.0)	1,216.6 (1,500.0)
Temperature	Mean T warm season (°C)	19.1 (0.4)	22.3 (2.1)	15.4 (0.5)	20.3 (0.1)	12.9 (0.3)	15.7 (1.8)	14.7 (0.4)	15.4 (0.4)	15.3 (0.1)
	SD of T warm season (°C)	4.6 (0.2)	4.1 (0.6)	4.1 (0.6)	4.7 (0.2)	5.5 (0.1)	4.5 (0.1)	4.4 (0.1)	4.1 (0.4)	4.5 (0.1)
	Mean T cool season (°C)	9.1 (1.4)	12.4 (3.6)	4.4 (0.5)	10.2 (0.7)	−2.7 (0.5)	4.3 (1.7)	−1.6 (0.8)	4.3 (0.3)	2.6 (0.1)
	SD of T cool season (°C)	4.2 (0.2)	4.2 (0.6)	5.6 (0.5)	4.4 (0.3)	6.0 (0.3)	6.5 (0.3)	6.2 (0.7)	5.6 (0.2)	6.1 (0.1)

BC, black carbon; CEANS, cardiovascular effects of air pollution and noise in Stockholm study; EPIC-NL, the European Prospective Investigation into Cancer and Nutrition-Netherlands; KORA, cooperative health research in the Augsburg region; NDVI, normalized difference vegetation index; NO_2_, nitrogen dioxide; O_3_, ozone during warm months (April to September); PM_2.5_, particulate matter with an aerodynamic diameter of less than 2.5 µm; T, temperature; SD, standard deviation; warm, months from April to September; cool, months from January to March and October to December).

### 3.1 Single-exposure effects

**Figure 1:**
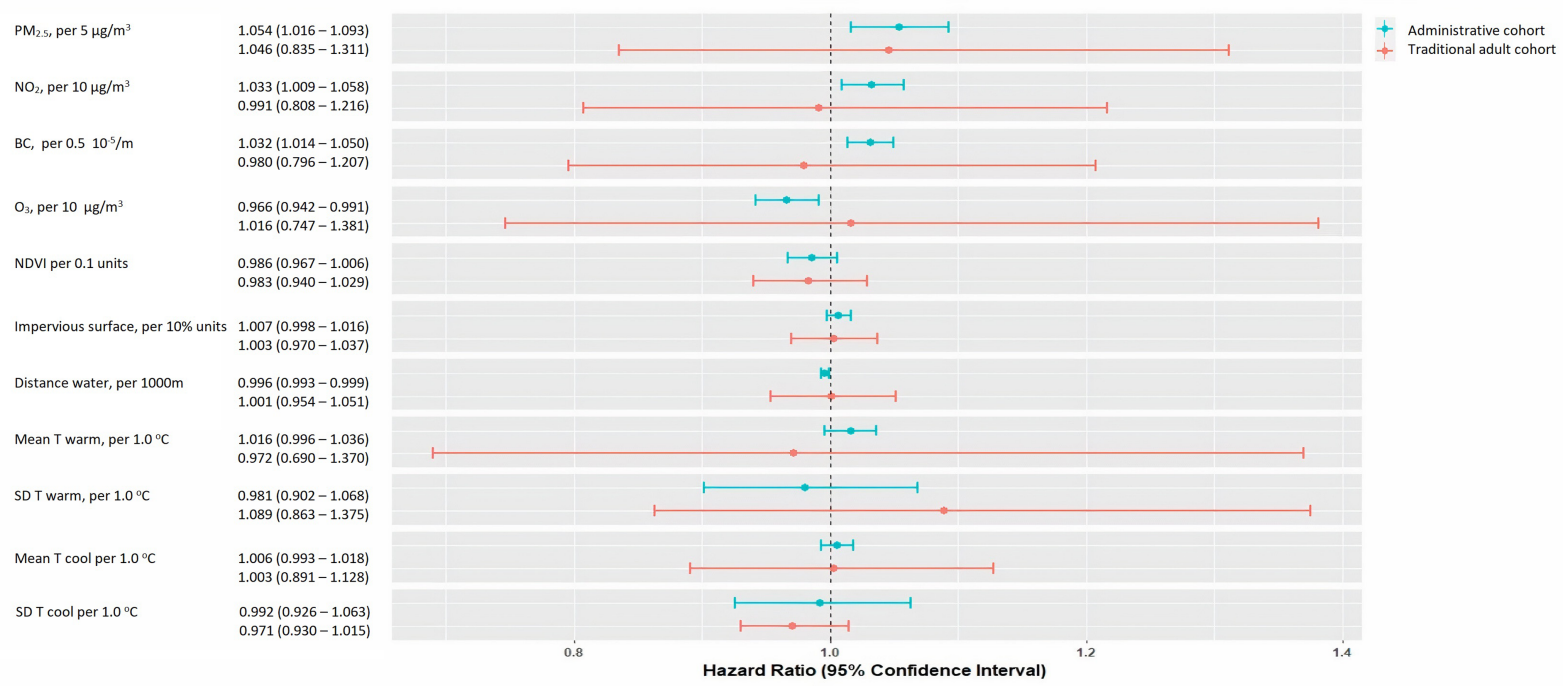
Pooled hazard ratios (HRs) and 95% confidence intervals (CIs) for the association between single exposures and all-cause mortality, by cohort type (administrative and traditional adult). BC, black carbon; NDVI, normalized difference vegetation index; NO_2_, nitrogen dioxide; O_3_, ozone during warm months (April to September); PM_2.5_, particulate matter with an aerodynamic diameter <2.5 µm; m, meters; temperature; SD, standard deviation; warm, months from April to September; cool, months from January to March and October to December). The availability of the covariates differs between cohorts. HRs are expressed per fixed increment and are adjusted for in the following: Catalonia: age (timescale), sex (strata) and county, smoking status, individual income, PSCA index, percentage of non-Spanish residents in census tract, and population density per m^2^; Greece: age (timescale), sex, NUTS1 areas country-wide (four levels: Attica/Aegean Islands, Crete/North Greece/Central Greece), tertiary education rate, unemployment rate, degree of urbanicity, and married rate. For the Greater Area of Athens and other large municipalities (population greater than 100,000 inhabitants) in Greece, the aforementioned variables were available at the square-block level. For the rest of the areas in Greece, the variables were available at the municipality unit level; the Netherlands: age (timescale), sex (strata), area, wealth in 2010—categorized in deciles, partner status in 2010, individual socioeconomical status, area-level socioeconomic status, area-level mean income in 2010, percentage of low-income households, urbanicity; Rome: age (timescale), sex (strata), place of birth, education, employment status, marital status, citizenship, deprivation index on a census block level and unemployment rate, percentage of graduates and house prices on a neighborhood level; Sweden: age (timescale), sex (strata), living conditions, level of education, district mean income, portion of people with high school or higher education in district, area; Switzerland: age (timescale), sex (strata), Swiss region (*n* = 7), marital status, occupational status, origin (i.e., Swiss vs. other), language region, socioeconomic position index (SEP), community-level SEP index; and community-level unemployment rate; CEANS: subcohort (strata), age (timescale), sex (strata), year of baseline visit, marital status, body mass index (BMI), smoking (status, duration, intensity, intensity squared), employment status, education, area-level socioeconomic status (2001 mean income on a neighborhood level); EPIC-NL: subcohort (strata), age (timescale), sex (strata), year of baseline visit, marital status, body-mass index, smoking (status, duration, intensity, intensity squared), employment status, education, area-level socioeconomic status (2001 mean income on a neighborhood level); KORA: subcohort (strata), age (timescale), sex (strata), and year of baseline visit, marital status, BMI, smoking (status, duration, intensity, intensity squared), employment status, education, area-level socioeconomic status (percentage of households with low income per 5 km^2^ grid cell in 2007).

Figure 1 presents the pooled HRs (and corresponding 95% CIs) of the association between all-cause mortality and single exposures. In the administrative cohorts, increased exposure to PM_2.5_, NO_2_, and BC was associated with all-cause mortality [pooled HRs: 1.054 (95% CI 1.016–1.093, *I*^2^ = 98%); 1.033 (95% CI 1.009–1.058, *I*^2 ^= 99%); and 1.032 (95% CI 1.014–1.050, *I*^2 ^= 98%), respectively]. Increased impervious surface and mean cool and warm season temperatures were associated with a higher risk of all-cause mortality, while increased O_3_, NDVI, distance to water, and temperature variation (in both the cool and warm seasons) were associated with a lower risk. Among the traditional adult cohorts, only the association between all-cause mortality and PM_2.5_ [HR: 1.046 (95% CI 0.835–1.311, *I*^2 ^= 0%)], impervious surface [HR: 1.003 (95% CI 0.970–1.037, *I*^2 ^= 36%)], warm temperature variation [HR: 1.089 (95% CI 0.863–1.375, *I*^2 ^= 0%)], and mean cool temperature [HR: 1.003 (95% CI 0.891–1.128, *I*^2 ^= 0%)] indicators were found to be adverse, however, not statically significant.

Cohort-specific and pooled HRs for the association under the increasing confounding adjustment in different models are presented in the Supplementary Material (Appendix V, Table S3). There was large heterogeneity between administrative cohorts for all exposures (*I*^2^* *> 81%; *p* < 0.01). Heterogeneity was also observed in most exposures between traditional adult cohorts (*I*^2^ range 0%–59%). Forest plots for the associations (based on main model 3) between all-cause mortality and single exposures are given in the Supplementary Material (Appendix V, Table S3 and Figures S1–S22).

### 3.2 Synergistic effects of NDVI and air pollution: warm season temperature

**Figure 2:**
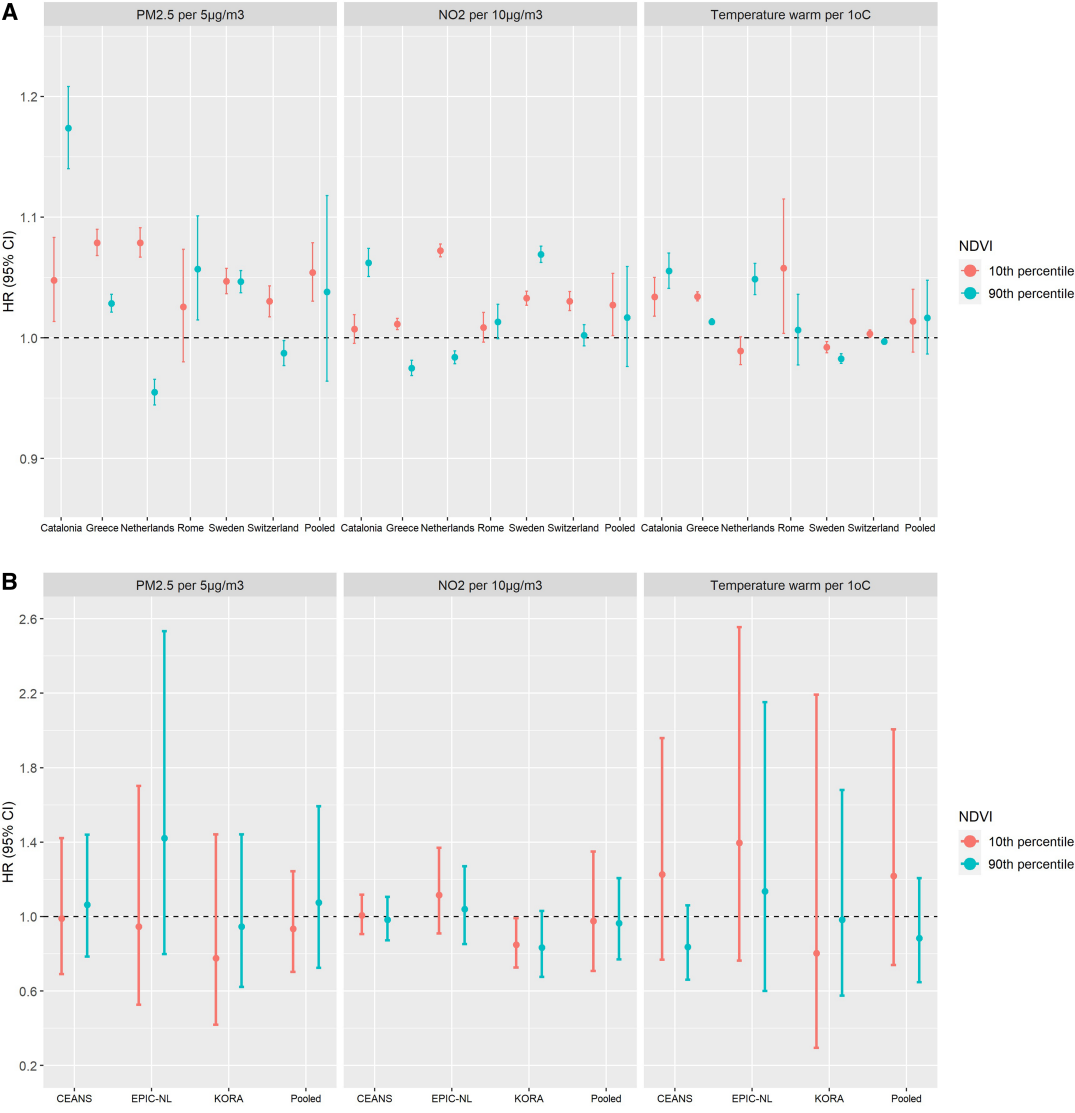
Cohort-specific and pooled hazard ratios^1^ (HRs) with corresponding 95% confidence intervals (CIs) for the interaction between NDVI and PM_2.5_, NO_2_, or mean temperature in the warm season (April to September), by cohort type (administrative and traditional adult). (**A**) Administrative cohorts. (**B**) Traditional adult cohorts. ^1^The HRs and corresponding 95% CIs for the interaction between air pollutants (PM_2.5_ or NO_2_) or mean temperature during the warm season and NDVI were calculated as an increment per 5 µg/m^3^ in PM_2.5_, 10 µg/m^3^ in NO_2_, or 1°C in mean warm temperature at the 10th percentile of NDVI distribution (as to assess the effect when exposed to low greenness) compared to the same increment in air pollutants and temperature exposures and the 90th percentile of NDVI distribution (for assessing the effect when exposed to high greenness). BC, black carbon; CEANS, cardiovascular effects of air pollution and noise in the Stockholm study; EPIC-NL, the European Prospective Investigation into Cancer and Nutrition-Netherlands; KORA, cooperative health research in the Augsburg region; NDVI, normalized difference vegetation index; NO_2_, nitrogen dioxide; O_3_, ozone during warm months (April to September); PM_2.5_, particulate matter with an aerodynamic diameter of less than 2.5 µm; m, meters; T, temperature; SD, standard deviation; warm, months from April to September; cool, months from January to March and October to December). HRs are expressed per fixed increment. Catalonia: age (timescale), sex (strata) and county, smoking status, individual income, PSCA index, percentage of non-Spanish residents in census tract, and population density per m^2^. Greece: age (timescale), sex, NUTS1 areas country-wide (four levels: Attica/Aegean Islands, Crete/North Greece/Central Greece), tertiary education rate, unemployment rate, degree of urbanicity, and married rate. For the Greater Area of Athens and other large municipalities (population greater than 100,000 inhabitants) in Greece, the aforementioned variables were available at the square-block level. For the rest of the areas in Greece, the variables were available at the municipality unit level. Netherlands: age (timescale), sex (strata), area, wealth in 2010 – categorized in deciles, partner status in 2010, individual socioeconomical status, area-level socioeconomic status, area-level mean income in 2010, percentage of low-income households, urbanicity. Rome: age (timescale), sex (strata), place of birth, education, employment status, marital status, citizenship, deprivation index on a census block level and unemployment rate, percentage of graduates, and house prices on a neighborhood level. Sweden: age (timescale), sex (strata), living conditions, level of education, district mean income, proportion of people with high school or higher education in district, area. Switzerland: age (timescale), sex (strata), Swiss region (*n* = 7), marital status, occupational status, origin (i.e., Swiss vs. other), language region, socioeconomic position index (SEP), community-level SEP index, and community-level unemployment rate. CEANS: subcohort (strata), age (timescale), sex (strata), year of baseline visit, marital status, body mass index (BMI), smoking (status, duration, intensity, intensity squared), employment status, education, area-level socioeconomic status (2001 mean income on a neighborhood level). EPIC-NL: subcohort (strata), age (timescale), sex (strata), and year of baseline visit, marital status, body-mass index, smoking (status, duration, intensity, intensity squared), employment status, education, area-level socioeconomic status (2001 mean income on a neighborhood level). KORA: subcohort (strata), age (timescale), sex (strata), year of baseline visit, marital status, body-mass index, smoking (status, duration, intensity, intensity squared), employment status, education, area-level socioeconomic status (percentage of households with low income per 5 km^2^ grid cell in 2007).

Figure 2A (administrative cohorts) and Figure 2B (traditional adult cohorts) present the cohort-specific and pooled results for the interaction between NDVI and (a) PM_2.5_, (b) NO_2_, and (c) mean temperature in the warm season. In the administrative cohorts, a lower NDVI (at the 10th percentile) seems to be significantly associated (interaction *p*-value <0.001) with a higher risk of all-cause mortality per 5 µg/m^3^ increase in PM_2.5_ and 10 µg/m^3^ increase in NO_2_ compared to a higher NDVI (effect at the 90th percentile). The pooled HRs for PM_2.5_ in areas with lower NDVI were 1.054 (95% CI 1.030–1.079, *I*^2 ^= 90%) vs. 1.038 95% CI 0.964–1.118, *I*^2 ^= 98%) in areas with a high NDVI. The corresponding HRs for NO_2_ were 1.027 (95% CI 1.002–1.053, *I*^2 ^= 98%) vs. 1.017 (95% CI 0.976–1.059, *I*^2 ^= 99%).

Forest plots of cohort-specific and pooled HRs for the interactions with NDVI are given in the Supplementary Material (Appendix VI, Figures S23–S34).

### 3.3 Multiple exposure models and cumulative risk index

**Figure 3:**
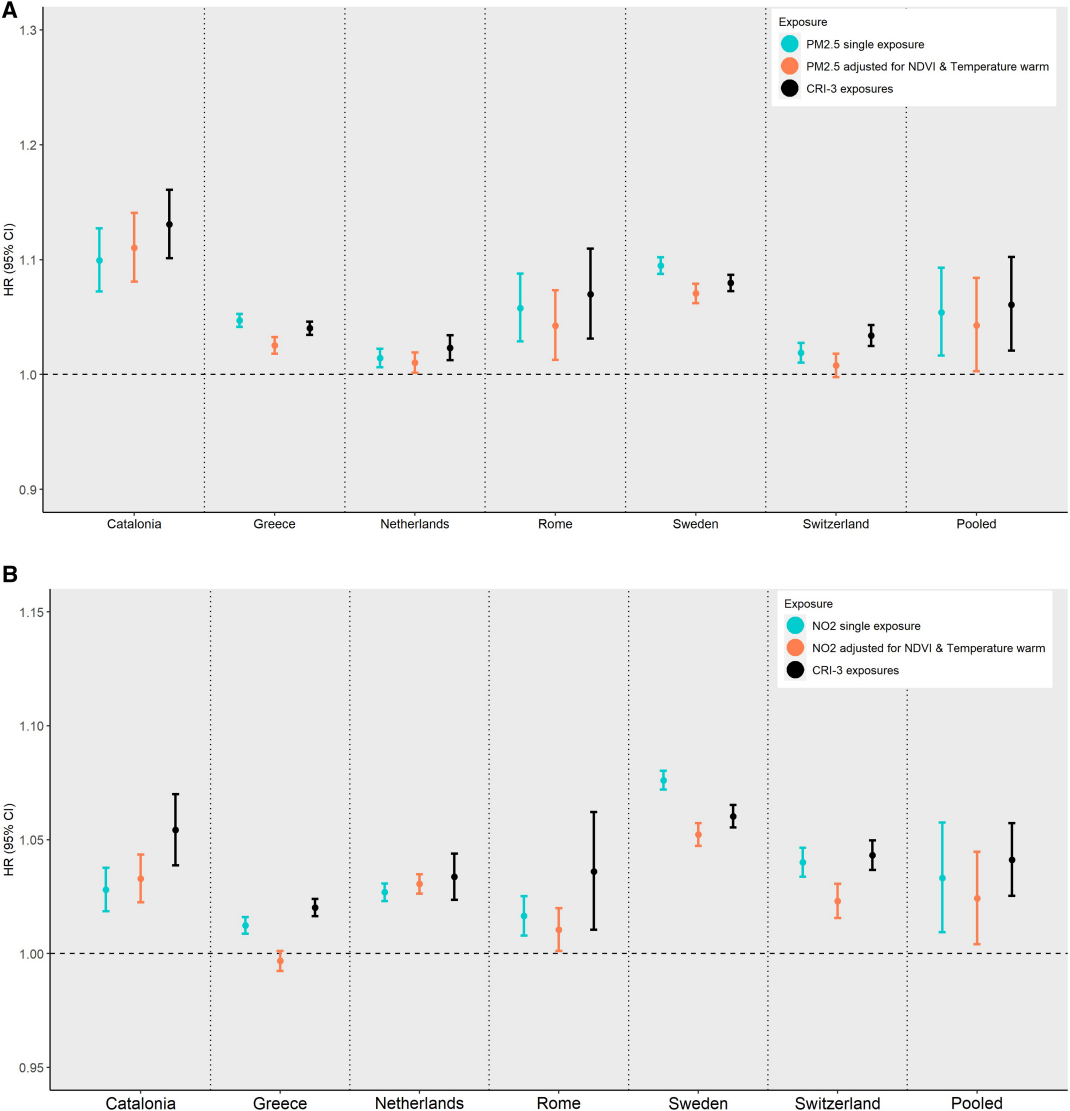
Cohort-specific and pooled hazard ratios (HRs) with corresponding 95% confidence intervals (CIs) for the association of all-cause mortality with PM_2.5_ (**A**,**B**) or NO_2_ (**C**,**D**) from (1) the single-exposure model, (2) further adjusting for NDVI and mean warm temperature, and (3) the cumulative (joint) effect of the three exposures from (2), by cohort type (administrative and traditional adult). (**A**) Administrative cohorts; PM_2.5_. (**B**) Administrative cohorts; NO_2_. (**C**) Traditional adult cohorts; PM_2.5_. (**D**) Traditional adult cohorts; NO_2._

Figure 3A–D summarizes the cohort type-specific pooled results for the association of all-cause mortality with PM_2.5_ (Figure 3A, B) or NO_2_ (Figure 3C, D) from (1) the single-exposure model, (2) after adjustment for NDVI and mean temperature in the warm season, and (3) the CRI of the three exposures, i.e., PM_2.5_ or NO_2_ plus NDVI plus mean temperature in the warm season, from the different exposures domains. The selection of the exposures from each domain was guided by the BIC in the single-exposure model to identify the optimal exposure per domain of air pollution, land/built environment, and ambient temperature. In the administrative cohorts, the pooled effect of PM_2.5_ or NO_2_ was slightly decreased when adjusting for NDVI and mean warm season temperature. For PM_2.5_, the pooled HR decreased from 1.054 (95% CI 1.016–1.093, *I*^2 ^= 98%) to 1.043 (95% CI 1.003–1.084, *I*^2 ^= 97%) when further adjusting for NDVI and mean temperature during the warm season. For NO_2_, the corresponding results showed a decrease in the pooled HR from 1.033 (95% CI 1.009–1.058, *I*^2 ^= 99%) to 1.024 (95% CI 1.004–1.045, *I*^2 ^= 98%). However, the joint effect of all three exposures, assessed through the CRI, resulted in the highest HR (PM_2.5_ pooled HR: 1.061, 95% CI 1.021–1.102, *I*^2 ^= 97%; and NO_2_ pooled HR: 1.041, 95% CI 1.025–1.057, *I*^2 ^= 97%). The magnitude of the pooled CRI in the traditional adult cohorts was similar but did not reach the nominal level of statistical significance. Forest plots are given in the Supplementary Material (Appendix VII, Figures S35–S42). No multicollinearity was present in any multiexposure models since VIFs <5 (data not shown).

**Figure:**
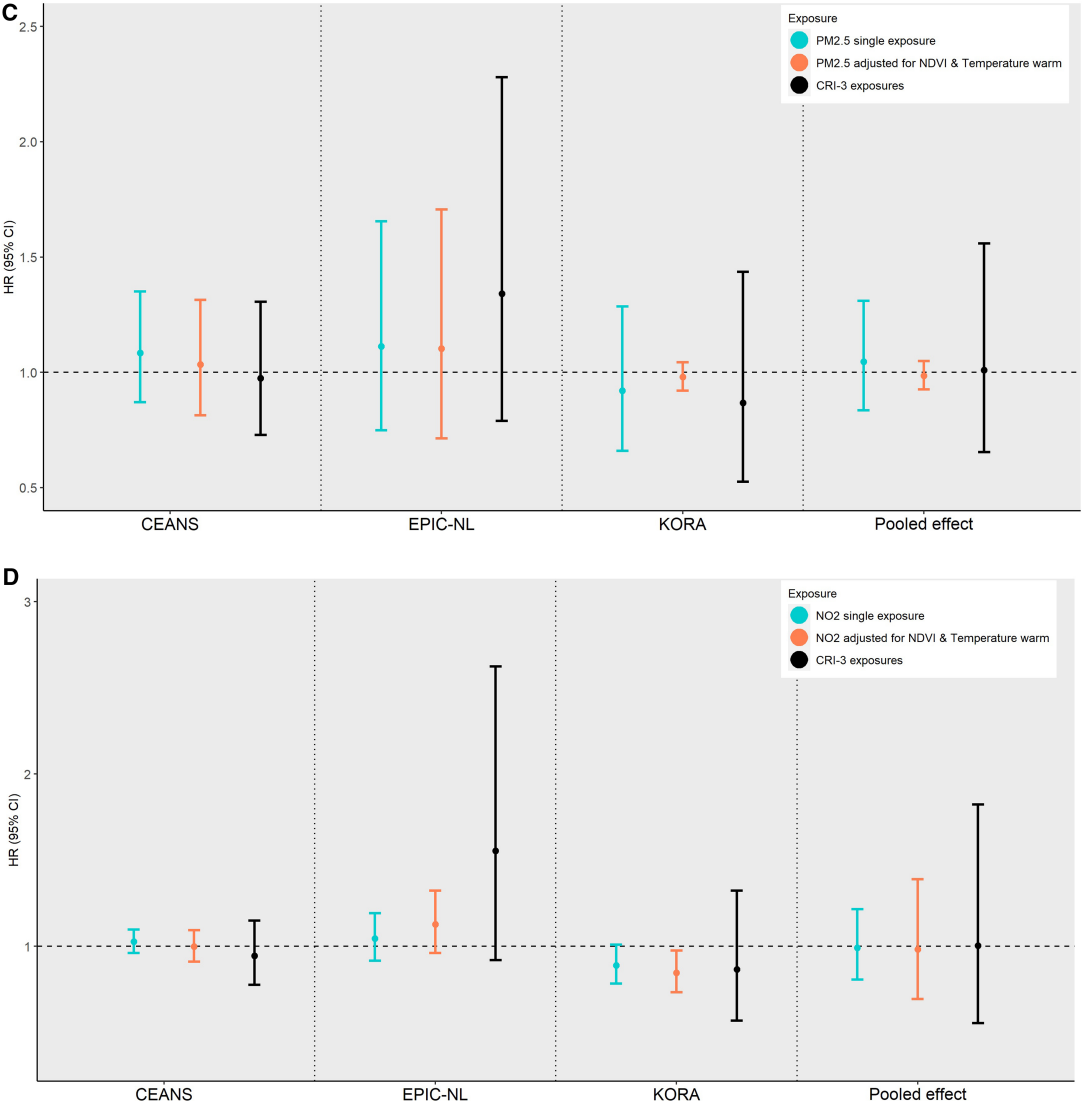


**Figure 4:**
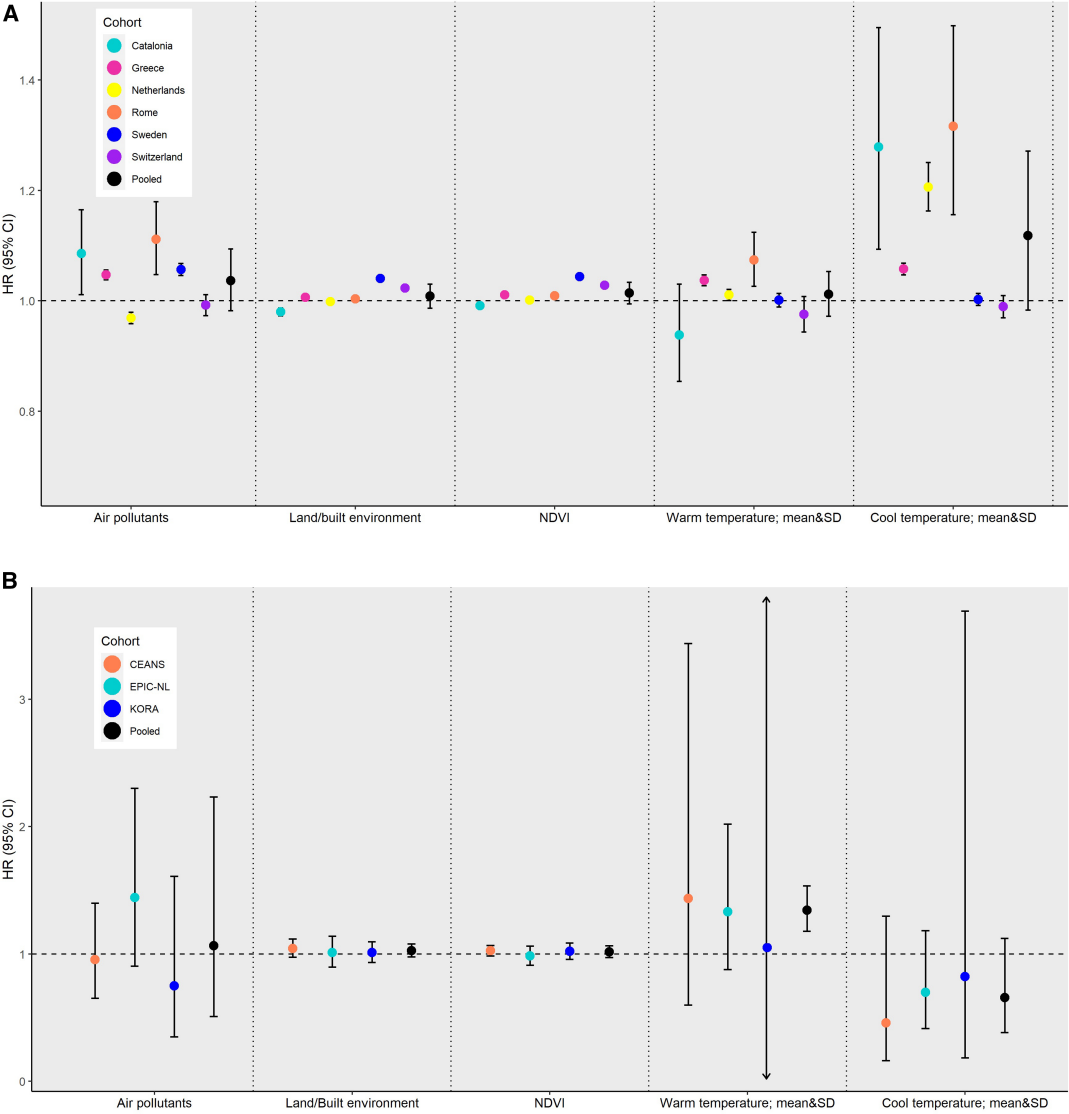
Cohort-specific and pooled hazard ratios (HRs) with corresponding 95% confidence intervals (CIs) for the cumulative (joint) effect on all-cause mortality by exposure domain and cohort type (administrative and traditional adult). (**A**) Administrative cohorts. (**B**) Traditional adult cohorts.

Figure 4 presents the cohort type-specific pooled CRI on all-cause mortality for the joint effect of all exposures within the same domain. In the administrative cohorts, the cumulative risk HR for all domains indicated an increased risk for all-cause mortality but did not reach the nominal level of statistical significance. The magnitude of the pooled cumulative effect in the traditional adult cohorts was similar. Note that the reported CRI in the land/built environment and NDVI was assessed per 0.1 unit decrease in the NDVI. Forest plots of cohort-specific and pooled CRI HRs from same domain exposures are given in the Supplementary Material (Appendix VIII, Figures S43–S52).

## 4 Discussion

We report results from a large multicenter European study under the EXPANSE project, including six large administrative cohorts and three traditional adult cohorts, on the long-term effects of multiple exposures on all-cause mortality. We investigated the effects of long-term single- and multiexposures to modeled air pollutant concentrations (PM_2.5_, NO_2_, BC, and O_3_ warm season), land/built environment characteristics defined by NDVI, impervious surfaces and distance to water, and to increased or decreased ambient temperature (annual mean and standard deviation during the warm and cool season) at the residential address of 27,731,158 individuals across Europe on all-cause mortality. We report consistent effects of PM_2.5_, NO_2_, and BC exposure on all-cause mortality. Synergistic effects of NDVI and PM_2.5_ and NO_2_ were observed; lower NDVI values were associated with a higher risk of all-cause mortality associated with increases in PM_2.5_ or NO_2_ compared to associations observed in higher NDVI areas. The joint effect, assessed through the CRI, of selected exposures to represent each domain was stronger compared to single-exposure effects. In the traditional cohorts, the results did not reach the nominal level of significance.

### 4.1 Single-exposure effects

#### 4.1.1 Air pollution

In the administrative cohorts, long-term exposure to air pollution was significantly associated with all-cause mortality in single-exposure models. The combined HRs across the six administrative cohorts were 1.054 per 5 µg/m^3^ increase in PM_2.5_, 1.033 per 10 µg/m³ NO_2_, and 1.032 per 0.5 × 10^−5^/m BC. These findings are similar to those observed in the ELAPSE study, in which data from seven administrative cohorts (aged ≥30 years) from Belgium, Denmark, England, the Netherlands, Norway, Rome (Italy), and Switzerland were analyzed using the same method of exposure assessment (43). In ELAPSE, significant associations were reported for non-accidental mortality and PM_2.5_, NO₂, and BC, with a HR of 1.053 (95% CI 1.021–1.085) per 5 µg/m³ increment in PM_2.5_, 1.044 (95% CI 1.019–1.069) per 10 µg/m³ increase in NO_2_, and 1.039 (95% CI 1.018–1.059) per 0.5 × 10^−5^/m in BC. Two of the ELAPSE administrative cohorts are also included in our analysis (i.e., the Netherlands and the Rome cohorts) and yielded similar findings considering that the analysis period and confounding adjustment differed between projects. The pooled effect estimates from the three traditional adult cohorts in our study were lower and did not reach statistical significance; in contrast to the combined results across eight traditional adult cohorts reported in Strak et al. (4) [per 5 µg/m^3^ in PM_2.5_ HR: 1.130 (95% CI 1.106–1.155); per 10 µg/m^3^ in NO_2_ HR: 1.086 (95% CI 1.070–1.102); per 0.5 × 10^−5^/m in BC HR: 1.081 (95% CI 1.065–1.098)]. A recent meta-analysis of 25 studies by Chen and Hoek (2) reported a risk ratio (RR) for PM_2.5_ and natural-cause mortality of 1.08 (95% CI 1.06–1.09) per 10 µg/m^3^ worldwide; while for NO_2_, a meta-analysis of 24 studies by Huangu and Atkinson (3) reported a RR of 1.02 (95% 1.01–1.04) per 10 µg/m^3^ increase.

We observed significant heterogeneity in almost all analyses driven by the magnitude of the effects (and not the small CIs, e.g., in the administrative cohorts). The lack of power in the analysis of the three traditional cohorts prevents the comparison with the administrative cohorts' analysis, although it strengthens the confidence in case of consistency in the direction as was the case with PM_2.5_ and NDVI.

#### 4.1.2 Land/built environment

Green spaces in the urban environment have been associated with improved health, specifically for mental and cardiovascular outcomes (14, 44, 45). Only a few studies have investigated the association between exposure to greenness and all-cause or non-accidental mortality. The pooled effect estimates in the present study [administrative cohorts HR: 0.986 (95% CI 0.967–1.006); traditional adult cohorts HR: 0.983 (95% CI 0.940–1.029)] are lower compared to those reported in a recent meta-analysis (20) of nine cohort studies, where a 4% decrease was observed per 0.1 unit increment of NDVI. Another meta-analysis that included eight cohort studies in individuals aged ≥60 years indicated that a 0.1 unit increase in NDVI was associated with a decreased risk of all-cause mortality by 1% (46). To our knowledge, only one meta-analysis has investigated the effect of urban blue spaces on health (19) and reported a 1.4% decrease in all-cause mortality for exposure vs. non-exposure that is comparable to our finding [administrative cohorts HR per 1,000 m distance from water: 0.996 (95% CI 0.993–0.999)]. The increasing built environment in urban areas (i.e., increased percentage of impervious surfaces) basically represents the opposite effect to exposure to green spaces, as indicated by their strong inverse correlation, and appears to have negative effects on health mostly in the analyses of the administrative cohorts.

#### 4.1.3 Ambient temperature

No clear associations were observed when using different metrics (mean and standard deviation) for long-term exposure to increased or decreased ambient temperature by season. Two recent systematic reviews indicated that long-term exposure to non-optimal temperatures may be associated with several health outcomes (10, 47). However, the number of identified studies was too small to allow for a meta-analysis.

### 4.2 Synergistic effects of NDVI and air pollution: warm season temperature

A limited number of epidemiological studies have reported on the potential synergistic effects between air pollution, temperature, and green spaces. Our results showed a significant interaction between NDVI and PM_2.5_ or NO_2_. The effect of PM_2.5_ or NO_2_ on all-cause mortality was stronger in areas with low levels of NDVI vs. areas with higher levels. Our findings are in line with previous relevant research. A population-based Canadian cohort (48) displayed a noticeable decrease in the effect of PM_2.5_ on mortality with increasing greenness within 500 m of their residences. In the Chinese Longitudinal Healthy Longevity Survey, Ji et al. (49) found a significant interaction between PM_2.5_ and NDVI. The research of long-term temperature effects on mortality has only just gained attention. Therefore, prior understanding on how long-term exposure to non-optimal temperature averages or variations affects health is limited. On the other hand, the health effects of short-term exposure to high or low temperatures are well established; Qiu et al. (50), in the Chinese Longitudinal Healthy Longevity Survey, reported that a higher level of surrounding greenness may be associated with a smaller temperature-related mortality risk at high temperatures. In particular, the findings suggest that individuals residing in the lowest quartile of NDVI compared to those residing in the highest quartile of NDVI had a 38% higher risk [RR: 1.38 (95% CI 0.79–2.42)] of mortality on days at the 95th percentile of temperature compared to days at the 50th percentile of temperature. Choi et al. (51) investigated the heat-mortality relationship among 452 locations with different levels of greenness (based on the Enhanced Vegetation Index (EVI)) in 24 countries. They found that cities with a high greenspace value had the lowest heat-mortality relative risk [RR: 1.19 (95% CI 1.13–1.25)], while the cities with a low greenspace value had a risk of 1.46 (95% CI 1.31–1.62) when comparing the 99th percentile of temperature and the minimum mortality temperature.

### 4.3 Multiple exposure models and cumulative risk index

Little is still known about the joint impact of multiple environmental exposures, such as air pollution, greenness, and ambient temperature on health outcomes. One of the novel contributions of our study is the investigation of the joint effects of multiple exposures, by estimating the CRI of multiple exposures within the same and across the different exposure domains (air pollution, land/built environment, ambient temperature) on all-cause mortality. We found that adjustment for NDVI and mean warm season temperature in the multiexposures across-domains models decreased the association for PM_2.5_ or NO_2_ and all-cause mortality. Indicatively, the pooled HR per 5 µg/m^3^ increase in PM_2.5_ decreased from 1.054 to 1.043, while the cumulative HR for the three selected exposures (PM_2.5_, NDVI, and mean warm temperature) was higher, at 1.061.

In the administrative cohorts, the effects of air pollutants (PM_2.5_ and NO_2_), after adjusting for mean temperature during the warm season and NDVI, generally decreased but remained significant. On the other hand, the CRI of the three exposures was stronger than the individual effect of PM_2.5_ or NO_2_. These findings suggest that all exposures may have independent effects on all-cause mortality, but also indicate that the risk of mortality is increased when taking into account the whole deteriorating surrounding environment: exposure to increased air pollution; decreased surrounding greenness; and increased temperature during the warm season. A similar pattern was observed in a national-level cohort in Canada, where Crouse et al. (52) reported significant, positive associations of single- and multiair pollutant exposures on non-accidental deaths. Individually, PM_2.5_ and NO_2_ were associated with deaths from non-accidental causes [HR: 1.035 (95% CI 1.029–1.041) and 1.052 (95% CI 1.045–1.059), respectively]. When mutually adjusting for the other two pollutants, the pollutant-specific effect was lower, but the joint effect of PM_2.5_, NO_2_, and O_3_ was stronger [HR: 1.075 (95% CI 1.067–1.084)].

### 4.4 Strengths and limitations

Our study had increased statistical power to study the targeted associations due mainly to the large sample sizes of the administrative cohorts across Europe. Six administrative cohorts and three traditional adult cohorts, with a total of 27,788,811 participants, 3,138,309 cases, and more than 204 million person-years at risk, were included in the present analysis. Moreover, exposures to multiple environmental stressors were harmonized between cohorts and derived using spatially resolved European-wide exposure surfaces. Due to data protection, all cohort-specific data were analyzed locally and in a national secure environment for the administrative cohorts. However, all statistical analyses were conducted under a common statistical protocol and by applying R codes that were developed centrally and distributed to all analysts.

Even though the present study applied a harmonized methodology, the presence of a high degree of heterogeneity in the results from the administrative cohorts must be underlined. Four out of six administrative cohorts were country-wide and included populations also residing in rural areas compared to the traditional adult cohorts, where participants live in urban areas. Moreover, we observed some differences in the results derived from traditional adult and administrative cohorts in the same country. Apart from the varying degree of area urbanization, this may be due to differences in the exposures, such as the chemical composition of particles (53). A limitation of our study is the small number of individual-level lifestyle covariates available in administrative cohorts that we compensate by including several area-level covariates. For example, in the Greek administrative cohort, we accounted only for age and sex and further included four area-level covariates. Nevertheless, the ELAPSE and MAPLE studies have indicated that further adjustment for smoking and body mass index using an indirect approach did not affect the effect estimates of the analysis of the administrative cohorts (41–43). However, there is always the potential for residual confounding. Other limitations of our study may be considered, including the varying timelines of the follow-up periods between the administrative and traditional adult cohorts and the time lag of the exposure associated with the outcome. However, previous findings indicate that the spatial contrasts over longer time periods, which are of interest in this present study, are stable. Studies in the Netherlands, Rome (Italy), the United Kingdom also for BC, and Vancouver (Canada) have reported that the spatial contrast of NO_2_ has been stable ≥10 years (54–57). de Hoogh et al. (58) documented the stability of measured and modeled spatial surfaces in Europe to at least the year 2000 for NO_2_ and O_3_ and documented the spatial robustness of the developed PM_2.5_, NO_2_, BC, and O_3_ LUR models on a continental (Western European) scale (36). In addition, other exposures, such as NDVI, are stable over time, depending on land-use changes that are usually slower paced. Vienneau et al. (59) found high address-level correlations (*r* = 0.89) between the years 2000 and 2014. As the time lag of the exposure associated with the outcome is also related to the impact of measurement error in the exposures on the effect estimates, previous research has indicated that results are mostly biased towards the null (60, 61). Regarding temporal trends in air pollution levels, the multicenter European ESCAPE study on the effects of long-term exposure to air pollution on mortality (62) showed that back-extrapolated NO_2_ and PM_10_ concentrations at baseline in the year of recruitment (mid-1990s) and the concentrations based on the 2008–2011 measurement campaign resulted in essentially the same HRs. Even though we assessed 11 different exposures from three different domains, noise exposure was not available at the present time. Exposure to noise has been linked to a series of non-auditory health outcomes, such as annoyance, sleep disturbance, stress, hypertension, cardiovascular disease, and poor mental health (63). However, evidence of road-traffic–related noise exposure effects on all-cause mortality has not yet been thoroughly addressed. A meta-analysis of five related studies published between 2000 and 2020 found that an increase per 10 dB in exposure to road-traffic–related noise was associated with a pooled risk [RR: 1.01 (95% CI 0.98–1.05)] for natural-cause mortality (64). The independent and joint effects of noise exposure on all-cause mortality should be considered in future research. Our analysis does not account for time-activity patterns as we only assessed residential exposure, which has been shown to reflect individual exposure satisfactorily. Finally, future investigation of time-varying annual exposures (which were not available for our analysis) may elaborate on the most relevant exposure window, especially for the analysis of less studied exposures, such as long-term increased or decreased temperature. Future research is needed to elaborate on the pathogenesis of environmental stressors in cause-specific mortality.

To the best of our knowledge, previous epidemiological studies have not addressed the joint effects of exposure to air pollution, greenness, and temperature conditions on health. In 2021, the WHO tightened its Air Quality Guidelines (65). The re-evaluation of the previous limit values (66) reflected the strong evidence provided by studies on air pollution-related health effects. In addition, the combined role of multiple environmental exposures on mortality is of great importance for supporting public health policy strategies. Therefore, apart from taking action to further reduce air pollution levels, the results from joint exposures could inform evidence-based policy on interventions, such as the management of green spaces and adaptation strategies for the climate, in order to address the public health burden from environmental stressors and particularly the effects of air pollution exposure.

In conclusion, the wealth of the exposure and health data in the EXPANSE project provide a powerful tool to investigate the complex associations between environmental exposures and mortality. The findings of our study support the consistent independent effects of long-term exposure to air pollution and greenness, but also highlight the increased effect of air pollution when interplaying with other environmental exposures.

## Data Availability

The cohort datasets presented in this article are not available because the cohort data could not be shared among the EXPANSE project members including named authors, nor can the data be shared externally due to strict national data protection regulations and the General Data Protection Regulation of the EU. The exposure maps are available on request from KdH (c.dehoogh@swisstph.ch).
