# Supplementary material for: Disentangling associations between multiple environmental exposures and all-cause mortality: an analysis of European administrative and traditional cohorts
Source: Front Epidemiol. 2024 Jan 12;3:1328188. doi: 10.3389/fepid.2023.1328188 (PMC10910955; doi:10.3389/fepid.2023.1328188)
Supplement: Supplementary file 1 [file Datasheet1.docx]

Supplementary Material

**Disentangling associations between multiple environmental exposures and all-cause mortality: an analysis of European administrative and traditional cohorts**

**Konstantina Dimakopoulou^1*^, Federica Nobile^2 3^, Jeroen de Bont^3^, Kathrin Wolf^4^, Danielle Vienneau^5 6^, Dorina Ibi^7^, Fabián Coloma^8^, Regina Pickford^4^, Christofer Åström^9^, Johan Nilsson Sommar^9^, Maria-Iosifina Kasdagli^1^, Kyriakos Souliotis^10 11^, Anastasios Tsolakidis^12^, Cathryn Tonne^8 13 14^,**

*** Correspondence:** Konstantina Dimakopoulou [kdimakop@med.uoa.gr](mailto:kdimakop@med.uoa.gr)

^1^ Department of Hygiene, Epidemiology and Medical Statistics, Medical School, National and Kapodistrian University of Athens, Athens, Greece

^2^ Department of Epidemiology, Lazio Region Health Service / ASL Roma 1, Rome, Italy

^3^ Institute of Environmental Medicine, Karolinska Institutet, Stockholm, Sweden

^4^ Institute of Epidemiology, Helmholtz Zentrum München, German Research Center for Environmental Health, Neuherberg, Germany

^5^ Swiss Tropical and Public Health Institute Basel, Allschwil, Switzerland

^6^ University of Basel, Basel, Switzerland

^7^ Institute for Risk Assessment Sciences (IRAS), Utrecht University, Utrecht, the Netherlands

^8^ ISGlobal, Barcelona, Spain

^9^ Department of Public Health and Clinical Medicine, Umeå University, Sweden

^10^ Department of Social and Education Policy, University of Peloponnese, Corinth, Greece

^11^ Health Policy Institute, Athens, Greece

^12^ IDIKA SA - e-Government Center for Social Security Services, Athens, Greece.

^13^ Universitat Pompeu Fabra (UPF), Barcelona, Spain

^14^CIBER Epidemiología y Salud Pública (CIBERESP), Madrid, Spain

^15^ Department of Clinical Sciences and Education, Södersjukhuset, Karolinska Institutet, Stockholm, Sweden

^16^ Sachś Children and Youth Hospital, Södersjukhuset, Stockholm, Sweden

^17^ Department of Cardiology, Danderyd Hospital, Stockholm, Sweden

^18^MRC Centre for Environment and Health, Environmental Research Group, Imperial College London, United Kingdom NIHR HPRU in Environmental Exposures and Health, Imperial College London, London, UK.

**Appendix I: Description of the cohorts**

**Catalonia administrative cohort**

The cohort was built through record-linkage using data collected in the public health administration databases of Catalonia. The public healthcare system covers nearly the entire population (98.8% of the 7.4 million in 2015). Catalonia (32,113 km^2^) is composed of 947 municipalities, grouped in seven health regions (median area 5,425 km2). The cohort includes individual-level (age, sex, comorbidities, smoking status, and income) information and is linked to small area-level socioeconomic data linked to the geocoded residential addresses. Area level SES was measured as PSCA Index, and indicator of deprivation where larger values indicate greater deprivation (https://www.idescat.cat/pub/?id=ist) as well as the proportion of non-Spanish residents at the census tract level in 2018. The original cohort included 5,127,059 adult (≥18 years) residents of Catalonia, who were covered by the public healthcare system in 2015.17 Follow- up in this analysis was until 31 December 2019. The cohort, and its predecessor, have been described in detail elsewhere. We received approval the Parc de Salut Mar Ethics Committee (CEIM-PS MAR, no. 2020/9610).

*Main references:*

Avellaneda-Gómez C, Vivanco-Hidalgo RM, Olmos S, Lazcano U, Valentin A, Milà C, et al. Air pollution and surrounding greenness in relation to ischemic stroke: A population-based cohort study. Environ Int. 2022;161(November 2021).

Ranzani O, Alari A, Olmos S, Milà C, Rico A, Ballester J, et al. Long-term exposure to air pollution and severe COVID-19 in Catalonia: a population-based cohort study. Nat Commun. 2023;14(1).

**Greece administrative cohort**

The Greek administrative cohort (GRAD, https://www.idika.gr/) is a nationwide population-based cohort. GRAD includes all adults with a social security number aged 37+years in 2014 and follows them until the end of 2019. The cohort contains data on disease diagnoses and related medication subscription and is linked with the mortality registry of the Hellenic Statistical Authority. The administrative cohort includes Individual-level (age, sex, education) information and is linked to small area-level (income, education, unemployment etc.) data from the census 20011 linked to the geocoded residential addresses. The study was approved by the Committee of Ethics and Research Ethics, National and Kapodistrian University of Athens and by the Bioethics Committee of Medical School, National and Kapodistrian University of Athens.

**Rome administrative cohort**

The Rome Longitudinal Study (RoLS) includes adults aged 30 years and older who were resident in Rome on October 9th, 2011 (the reference day of the Census). The population-based cohort was followed-up until 2018 and the subjects were linked to Health Information Systems available in the Lazio Region through an anonymous identification code. Individual-level (age, sex, marital status, occupation status, education, etc.) and area-level (deprivation index, unemployment rate, etc.) information, through geocoded residential addresses at baseline, is available for each subject. The enrolment and selecting procedures were similar to those described in more detail for the 2001 RoLS.

*Main references:*

Cesaroni G, Badaloni C, Romano V, Donato E, Perucci CA, Forastiere F. Socioeconomic position and health status of people who live near busy roads: the Rome Longitudinal Study (RoLS). Environ Health. 2010 Jul 21;9:41. doi: 10.1186/1476-069X-9-41. PMID: 20663144; PMCID: PMC2918588.

Cesaroni G, Badaloni C, Gariazzo C, Stafoggia M, Sozzi R, Davoli M, Forastiere F. Long-term exposure to urban air pollution and mortality in a cohort of more than a million adults in Rome. Environ Health Perspect. 2013 Mar;121(3):324-31.

Cesaroni G, Venturini G, Paglione L, Angelici L, Sorge C, Marino C, Davoli M, Agabiti N. Differenziali di mortalità a Roma: il ruolo dell’istruzione e dei prezzi immobiliari del quartiere di residenza [Mortality inequalities in Rome: the role of individual education and neighbourhood real estate market]. Epidemiol Prev. 2020 Sep-Dec;44(5-6 Suppl 1):31-37. Italian. doi: 10.19191/EP20.5-6.S1.P031.071. PMID: 33415944.

**Sweden administrative cohort**

The SIMSAM is a register based cohort with all adults in Sweden. For the study all individuals aged above 37 in 2010 was included. The cohort contain data on all individuals up until exclusion (incident, death, migration etc.) or the end of the follow-up period. Individual-level (age, sex, living condition, education, etc.) and area-level (income, education, etc.) information, through geocoded residential addresses for each year, is available for each subject. The Umeå SIMSAM Lab data infrastructure used in this study was developed with support from the Swedish Research Council, the Riksbanken Jubileumsfond and by strategic funds from Umeå University.

**Switzerland administrative cohort**

The Swiss National Cohort (SNC) is a national longitudinal research platform linking census data with birth, mortality, and emigration data. The SNC was approved by the Ethics Committees of the Cantons of Zurich and Bern. Due to mandatory participation, nearly all persons residing in Switzerland at the time of the 1990 and 2000 censuses are represented; an estimated 98.6% residents participated in 2000. For each person, the SNC contains an individual (e.g., sex, date of birth, occupation), household (e.g., type of household, socio-economic position (SEP)), and building (e.g., type of building, number of floors, geographical coordinate) record. Prior to 2010 the SNC was based on a probabilistic linkage. In 2010, Switzerland replaced the classic door-to-door census system with the registry-based census repeated each year. As such, a deterministic linkage with a unique pseudo-ID (SNC-ID), based on the social security number but cannot be traced back to it, is now used. In this new framework, data on education, occupation, employment or religion is only collected in an annual structural enquiry of a random sample of about 250,000 people per year. Swiss TPH received the latest SNC data (for 1990-2019) in July 2021 with all the necessary permissions to conduct analyses.

*Main references:*

Bopp, M., Spoerri, A., Zwahlen, M., Gutzwiller, F., Paccaud, F., Braun-Fahrlander, C., Rougemont, A., Egger, M., 2009. Cohort Profile: The Swiss National Cohort—a longitudinal study of 6.8 million people. Int. J. Epidemiol. 38 (2), 379–384.

Spoerri, A., Zwahlen, M., Egger, M., Bopp, M., 2010. The Swiss National Cohort: a unique database for national and international researchers. Int. J.Public Health. 55 (4), 239–242.

Renaud, A., 2004. Coverage Estimation for the Swiss Population Census 2000: Estimation Methodology and Results. Swiss Statistics Methodology Report. Swiss Federal Statistical Office, Neuchatel, p. 147.

Panczak, R., Galobardes, B., Voorpostel, M., Spoerri, A., Zwahlen, M., Egger, M., 2012. A Swiss neighbourhood index of socioeconomic position: development and association with mortality. J. Epidemiol. Comm. Health. 66 (12), 1129–1136.

**The Netherlands administrative cohort**

The selection of the Dutch national cohort has been described elsewhere (Fischer et al., 2015). In short, in the Netherlands population statistics based on digital municipal registers are combined by Statistics Netherlands (http://www.cbs.nl/en-GB/menu/home/default.htm) into a longitudinal file for each individual registered in the municipal registration. These records start on 1 January 1995. Changes in demographic attributes (e.g., death, residential address, partner status, emigration, region of origin) are updated yearly by adding additional information on the nature and the date of the change. In these files, the individual identification number is replaced by an encrypted unique identification number. This identification number is used to enrich the individual files with information from other central data sources available at Statistics Netherlands. For the EXPANSE study, we shifted the baseline to 1/1/20010 and follow-up to 31/12/2019, selecting all Dutch inhabitants who were 37 years of age or older at baseline. In addition, we included only individuals who lived at the same residential address since baseline and those who moved out of their baseline address but returned back within 92 days.

*Main references:*

P.H. Fischer, M. Marra, C.B. Ameling, G. Hoek, R. Beelen, K. de Hoogh, et al. Air pollution and mortality in seven million adults: the Dutch Environmental Longitudinal Study (DUELS) Environ. Health Perspect., 123 (2015), pp. 697-704

**CEANS cohort (Cardiovascular Effects of Air Pollution and Noise in Stockholm)**

The CEANS cohort is comprised of four subcohorts: The Screening Across the Lifespan Twin Study (SALT) sampled 7,043 individuals from the Swedish Twin Register born 1958 and earlier, who lived in Stockholm County (Lichtenstein et al. 2006). The Stockholm Diabetes Preventive Program (SDPP) is a population-based prospective study of 7,949 subjects aged 35–54 years (Erikssson et al. 2008). The SIXTY subcohort consists of a random population sample of one-third of all men and women living in Stockholm County turning 60 years between August 1997 and March 1999 (Wändell et al.2007). Lastly, The Swedish National Study of Aging and Care in Kungsholmen (SNAC-K) randomly sampled individuals 60+ years of age from a central area in Stockholm (Lagergren et al. 2004). All participants resided in Stockholm County, Sweden.

*Main references:*

Eriksson AK, Ekbom A, Granath F, et al. Psychological distress and risk of pre-diabetes and Type 2 diabetes in a prospective study of Swedish middle-aged men and women. Diabet Med 2008;25:834–42.

Lagergren M, Fratiglioni L, Hallberg IR, et al. A longitudinal study integrating population, care and social services data. The Swedish National study on Aging and Care (SNAC). Aging Clin Exp Res 2004;16:158–68.

Lichtenstein P, Sullivan PF, Cnattingius S, et al. The Swedish Twin Registry in the third millennium: an update. Twin Res Hum Genet 2006;9:875–82.

Wändell PE, Wajngot A, de Faire U, et al. Increased prevalence of diabetes among immigrants from non-European countries in 60-year-old men and women in Sweden. Diabetes Metab 2007;33:30–6.

**EPIC-NL cohort (European Prospective Investigation into Cancer and Nutrition, the Netherlands)**

The EPIC-NL combines two EPIC-cohorts within the Netherlands: The Monitoring Project on Risk Factors and chronic diseases in the Netherlands (MORGEN) cohort which consists of a general population sample aged 20–59 years from three Dutch towns (Amsterdam, Doetinchem and Maastricht). Prospect is a prospective cohort study among women aged 49–70, residing in the city of Utrecht or its vicinity, who participated in the nation-wide Dutch breast cancer screening programme between 1993 and 1997.

*Main reference:*

Beulens JWJ, Monninkhof EM, Verschuren WMM et al. Cohort Profile: The EPIC-NL study. International Journal of Epidemiology 2010; 39: 1170–78.

**KORA cohort** **(Cooperative Health Research in the Region of Augsburg)**

KORA is a regional research platform for population-based surveys and subsequent follow-up studies in the fields of epidemiology, health economics, and health care research (https://www.helmholtz-munich.de/en/epi/cohort/kora). This analysis includes two cross-sectional population-representative surveys that were conducted in 1994-1995 (S3 survey) and 1999-2001 (survey S4) in the city of Augsburg and two adjacent rural districts including inhabitants of German nationality aged 25 to 74. Follow-up was conducted in 2011 for S3 and 2013/14 for S4.

*Main reference:*

Holle R, Happich M, Lowel H, Wichmann HE. KORA--a research platform for population based health research. Gesundheitswesen 2005; 67 Suppl 1: S19-S25.

**Appendix II: Exposure assessment details**

**Table S1.** Data source, temporal resolution and time reference of the exposure factors grouped into the three domains: air pollution, land-built-environment and ambient temperature.

| **Domain** | **Exposure variable** | **Units** | **Data source** | **Temporal resolution** | **Time reference** |
| --- | --- | --- | --- | --- | --- |
| **Air pollution** | PM_2.5_, NO_2_, BC, O_3_ | µg/m^3^ | ELAPSE land-use regression model | Annual | 2010 |
| **Land-built environment** | NDVI | 0 to 1 | Terra Moderate Resolution Imaging Spectroradiometer (MODIS) with 250 m x 250 m | Annual | 2019 |
|  | Impervious surfaces | % | Copernicus Land Monitoring Service within 100mx100m grid cell | Annual | 2015 |
|  | Distance to blue spaces | meters | EU-Hydro map developed by the Copernicus Land Monitoring Service | - | 2011 |
| **Ambient temperature** | Mean temperature | Celsius | European Centre for Medium-Range Weather Forecasts (ECMWF) ERA5-Land | Summer and winter | 2010 |
|  | Standard deviation temperature | SD |  | Summer and winter | 2010 |

Abbreviations: BC, Black carbon; NDVI, Normalized Difference Vegetation Index; NO_2_, nitrogen dioxide; NO_x_, nitrogen oxides; O_3_, ozone during warm month; PM_2.5_, particulate matter with an aerodynamic diameter of less than 2.5 μm; SD, Standard deviation

**Appendix III: Individual- and area- level covariates included in each model, by cohort**

**Administrative cohorts**

Catalonia

Model 1: age (time scale), sex (strata) and county.

Model 2: additionally adjusted for smoking status, individual income.

Model 3: additionally adjusted for psca index, percentage of non-Spanish residents in census tract, and population density per m^2^.

Greece

Model 1: age (time scale), sex, NUTS1 areas country-wide (4 levels: Attica / Aegean Islands, Crete / North Greece / Central Greece).

Model 2: same as Model 1 since no other individual-level covariates were available.

Model 3: additionally adjusted for tertiary education rate, unemployment rate, degree of urbanicity and married rate. For the Greater Area of Athens and other large municipalities (population greater than 100,000 inhabitants) in Greece, the aforementioned variables were available at square-block level. For the rest of the areas in Greece, the variables were available at municipality unit level.

Netherlands:

Model 1: age (time scale), sex (strata), area.

Model 2: additionally adjusted for wealth at 2010, categorized in deciles, partner status at 2010, individual socioeconomical status.

Model 3; additionally adjusted for area-level socio-economic status, area-level mean income at 2010, percentage of low-income households, urbanicity.

Rome

Model 1: age (time scale), sex (strata).

Model 2: additionally adjusted for place of birth, education, employment status, marital status, citizenship.

Model 3: additionally adjusted for deprivation index on a census block level and unemployment rate, percentage of graduates and house prices on a neighbourhood level.

Sweden

Model 1: age (time scale), sex (strata).

Model 2: additionally adjusted for living condition, education level.

Model 3: additionally adjusted for district mean income, portion of people with high school or higher education in district, area.

Switzerland:

Model 1: age (time scale), sex (strata).

Model 2: additionally adjusted for Swiss region (n=7), marital status, occupational status,

origin (i.e. Swiss vs. other), language region, socio-economic position index (SEP).

Model 3: additionally adjusted for community-level SEP index and community-level unemployment rate.

**Traditional adult cohorts**

CEANS

Model 1: subcohort (strata), age (timescale), sex (strata), and year of baseline visit.

Model 2: additionally adjusted for marital status, body-mass index, smoking (status, duration, intensity, intensity squared), employment status, education.

Model 3: additionally adjusted for area-level socioeconomic status (2001 mean income on a neighbourhood level).

EPIC-NL:

Model 1: subcohort (strata), age (timescale), sex (strata), and year of baseline visit.

Model 2: additionally adjusted for marital status, body-mass index, smoking (status, duration, intensity, intensity squared), employment status, education.

Model 3: additionally adjusted for area-level socioeconomic status (2001 mean income on a neighbourhood level).

KORA

Model 1: subcohort (strata), age (timescale), sex (strata), and year of baseline visit.

Model 2: additionally adjusted for marital status, body-mass index, smoking (status, duration, intensity, intensity squared), employment status, and education.

Model 3: additionally adjusted for area-level socioeconomic status (percentage of households with low income per 5 km² grid cell in 2007).

**Appendix IV: Estimation of the Cumulative Risk Index (CRI)**

The product of the HRs based on the survival models including multi-exposures is interpreted as the cumulative risk estimate (CRI). This method has been used in similar previous research (Crouse et al. 2015). The estimated CRI is the additive effects of joint exposures on all-cause mortality. Thus, the CRI represents the relative hazard for fixed-unit change in multi-exposures compared with that for no change in any of the exposures. Let $x^{'}=\left( x_{1},\ldots,x_{m} \right)$ represent exposure level units of *m* exposures (i.e., PM_2.5_ μg/m^3^, NDVI units and mean temperature during the warm season ^o^C). The CRI is the HR based on the combination of the *m* exposures evaluated at x and is defined as:

$CRI=exp\left[ \sum_{m=1}^{m} \hat{\beta}_{m}x_{m} \right]\equiv exp\left( \hat{\beta}^{'}x \right)=\prod_{m=1}^{m} JHR_{m}$ ,

Where,

$\beta^{'}=\left( \beta_{1}^{'},\ldots,\beta_{m}^{'} \right)$ are the estimates of the log-hazard ratio for the *m* exposures estimated in the survival model consisting of all *m* exposures together and

${JHR}_{m}=exp\left( \beta_{m}^{'}x_{m} \right)$ denotes the cumulative (joint) hazard ratio for the m^th^ exposure in the multi-exposure survival model.

Further, we denote $Cov\left( \hat{\beta} \right)$ as the variance–covariance matrix of $\hat{\beta}$. The 95% confidence interval of CRI was defined by $exp\left[ \hat{\beta}^{'}x\pm1.96\times\hat{\beta}Cov\left( \hat{\beta} \right)\hat{\beta}^{'} \right]$.

**Reference**

Crouse DL, Peters PA, Hystad P, Brook JR, van Donkelaar A, Martin RV, Villeneuve PJ, Jerrett M, Goldberg MS, Pope CA 3rd, Brauer M, Brook RD, Robichaud A, Menard R, Burnett RT. Ambient PM2.5, O₃, and NO₂ Exposures and Associations with Mortality over 16 Years of Follow-Up in the Canadian Census Health and Environment Cohort (CanCHEC). Environ Health Perspect. 2015 Nov;123(11):1180-6

**Appendix V: Correlations between exposures, Single-exposure models, cohort specific and pooled results**

**Table S2.** Pearson correlation coefficient between exposure, in A. administrative and B. traditional adult cohorts.

**A. Administrative cohorts**

|  |  | **Exposure** | **Air pollution** | | | | **Built environment** | | | **Temperature** | | | |
| --- | --- | --- | --- | --- | --- | --- | --- | --- | --- | --- | --- | --- | --- |
| **Cohort** |  |  | NO_2_ (μg/m^3^) | PM_2.5_ (μg/m^3^) | BC  (10^5^/m) | O_3_ (μg/m^3^) | Distance water, m | Impervious surface, % | NDVI | Mean Ta cool (^o^C) | Mean Ta warm (^o^C) | SD Ta cool (^o^C) | SD Ta warm (^o^C) |
| **Catalonia** | **Air pollution** | NO_2_ (μg/m^3^) | 1 | 0.874 | 0.932 | -0.958 | 0.000 | 0.368 | -0.529 | 0.421 | 0.120 | -0.539 | -0.565 |
|  |  | PM_2.5_ (μg/m^3^) | 0.874 | 1 | 0.826 | -0.866 | -0.074 | 0.374 | -0.507 | 0.484 | 0.336 | -0.456 | -0.415 |
|  |  | BC (10^-5^/m) | 0.932 | 0.826 | 1 | -0.910 | 0.002 | 0.338 | -0.499 | 0.394 | 0.158 | -0.475 | -0.455 |
|  |  | O_3_ (μg/m^3^) | -0.958 | -0.866 | -0.910 | 1 | -0.037 | -0.307 | 0.461 | -0.358 | -0.094 | 0.471 | 0.519 |
|  | **Built environment** | Distance water, m | 0.000 | -0.074 | 0.002 | -0.037 | 1 | -0.148 | 0.138 | -0.068 | -0.079 | 0.033 | 0.017 |
|  |  | Impervious surface,% | 0.368 | 0.374 | 0.338 | -0.307 | -0.148 | 1 | -0.692 | 0.228 | 0.175 | -0.197 | -0.147 |
|  |  | NDVI | -0.529 | -0.507 | -0.499 | 0.461 | 0.138 | -0.692 | 1 | -0.313 | -0.239 | 0.285 | 0.214 |
|  | **Temperature** | Mean T cool (^o^C) | 0.421 | 0.484 | 0.394 | -0.358 | -0.068 | 0.228 | -0.313 | 1 | 0.779 | -0.831 | -0.599 |
|  |  | Mean T warm (^o^C) | 0.120 | 0.336 | 0.158 | -0.094 | -0.079 | 0.175 | -0.239 | 0.779 | 1 | -0.336 | 0.000 |
|  |  | SD T cool (^o^C) | -0.539 | -0.456 | -0.475 | 0.471 | 0.033 | -0.197 | 0.285 | -0.831 | -0.336 | 1 | 0.868 |
|  |  | SD T warm (^o^C) | -0.565 | -0.415 | -0.455 | 0.519 | 0.017 | -0.147 | 0.214 | -0.599 | 0.000 | 0.868 | 1 |
|  |  | **Exposure** | **Air pollution** | | | | **Built environment** | | | **Temperature** | | | |
|  |  |  | NO_2_ (μg/m^3^) | PM_2.5_ (μg/m^3^) | BC  (10^5^/m) | O_3_ (μg/m^3^) | Distance water, m | Impervious surface, % | NDV | Mean Ta cool (^o^C) | Mean Ta warm (^o^C) | SD Ta cool (^o^C) | SD Ta warm (^o^C) |
| **Greece** | **Air pollution** | NO_2_ (μg/m^3^) | 1 | 0.583 | 0.964 | 0.302 | 0.231 | 0.537 | -0.710 | 0.331 | 0.604 | -0.116 | 0.087 |
|  |  | PM_2.5_ (μg/m^3^) | 0.583 | 1 | 0.554 | -0.112 | -0.168 | 0.538 | -0.588 | 0.070 | 0.422 | 0.215 | 0.127 |
|  |  | BC (10^-5^/m) | 0.964 | 0.554 | 1 | 0.260 | 0.231 | 0.477 | -0.648 | 0.337 | 0.573 | -0.150 | 0.053 |
|  |  | O_3_ (μg/m^3^) | 0.302 | -0.112 | 0.260 | 1 | 0.306 | 0.068 | -0.194 | 0.678 | 0.599 | -0.475 | -0.305 |
|  | **Built environment** | Distance water, m | 0.231 | -0.168 | 0.231 | 0.306 | 1 | -0.030 | -0.010 | -0.024 | 0.109 | 0.074 | 0.197 |
|  |  | Impervious surface,% | 0.537 | 0.538 | 0.477 | 0.068 | -0.030 | 1 | -0.812 | 0.216 | 0.342 | -0.020 | -0.031 |
|  |  | NDVI | -0.710 | -0.588 | -0.648 | -0.194 | -0.010 | -0.812 | 1 | -0.337 | -0.526 | 0.088 | 0.002 |
|  | **Temperature** | Mean T cool (^o^C) | 0.331 | 0.070 | 0.337 | 0.678 | -0.024 | 0.216 | -0.337 | 1 | 0.759 | -0.772 | -0.651 |
|  |  | Mean T warm (^o^C) | 0.604 | 0.422 | 0.573 | 0.599 | 0.109 | 0.342 | -0.526 | 0.759 | 1 | -0.366 | -0.094 |
|  |  | SD T cool (^o^C) | -0.116 | 0.215 | -0.150 | -0.475 | 0.074 | -0.020 | 0.088 | -0.772 | -0.366 | 1 | 0.683 |
|  |  | SD T warm (^o^C) | 0.087 | 0.127 | 0.053 | -0.305 | 0.197 | -0.031 | 0.002 | -0.651 | -0.094 | 0.683 | 1 |
| **Netherlands** | **Air pollution** | NO_2_ (μg/m^3^) | 1 | 0.634 | 0.878 | -0.721 | -0.264 | 0.339 | -0.553 | 0.411 | 0.111 | -0.234 | -0.174 |
|  |  | PM_2.5_ (μg/m^3^) | 0.634 | 1 | 0.633 | -0.347 | -0.171 | 0.269 | -0.397 | 0.221 | 0.531 | 0.102 | 0.236 |
|  |  | BC (10^-5^/m) | 0.878 | 0.633 | 1 | -0.595 | -0.220 | 0.274 | -0.504 | 0.239 | 0.211 | -0.056 | 0.007 |
|  |  | O_3_ (μg/m^3^) | -0.721 | -0.347 | -0.595 | 1 | 0.256 | -0.188 | 0.372 | -0.386 | 0.058 | 0.301 | 0.293 |
|  | **Built environment** | Distance water, m | -0.264 | -0.171 | -0.220 | 0.256 | 1 | -0.156 | 0.228 | -0.198 | 0.039 | 0.160 | 0.170 |
|  |  | Impervious surface,% | 0.339 | 0.269 | 0.274 | -0.188 | -0.156 | 1 | -0.612 | 0.144 | 0.015 | -0.100 | -0.074 |
|  |  | NDVI | -0.553 | -0.397 | -0.504 | 0.372 | 0.228 | -0.612 | 1 | -0.235 | -0.056 | 0.141 | 0.104 |
|  | **Temperature** | Mean T cool (^o^C) | 0.411 | 0.221 | 0.239 | -0.386 | -0.198 | 0.144 | -0.235 | 1 | -0.190 | -0.872 | -0.726 |
|  |  | Mean T warm (^o^C) | 0.111 | 0.531 | 0.211 | 0.058 | 0.039 | 0.015 | -0.056 | -0.190 | 1 | 0.619 | 0.798 |
|  |  | SD T cool (^o^C) | -0.234 | 0.102 | -0.056 | 0.301 | 0.160 | -0.100 | 0.141 | -0.872 | 0.619 | 1 | 0.934 |
|  |  | SD T warm (^o^C) | -0.174 | 0.236 | 0.007 | 0.293 | 0.170 | -0.074 | 0.104 | -0.726 | 0.798 | 0.934 | 1 |
|  |  | **Exposure** | **Air pollution** | | | | **Built environment** | | | **Temperature** | | | |
|  |  |  | NO_2_ (μg/m^3^) | PM_2.5_ (μg/m^3^) | BC  (10^5^/m) | O_3_ (μg/m^3^) | Distance water, m | Impervious surface, % | NDVI | Mean Ta cool (^o^C) | Mean Ta warm (^o^C) | SD Ta cool (^o^C) | SD Ta warm (^o^C) |
| **Rome** | **Air pollution** | NO_2_ (μg/m^3^) | 1 | 0.785 | 0.906 | -0.791 | -0.192 | 0.390 | -0.492 | -0.189 | 0.234 | 0.305 | 0.355 |
|  |  | PM_2.5_ (μg/m^3^) | 0.785 | 1 | 0.661 | -0.677 | -0.198 | 0.335 | -0.386 | -0.314 | 0.187 | 0.423 | 0.479 |
|  |  | BC (10^-5^/m) | 0.906 | 0.661 | 1 | -0.823 | -0.142 | 0.411 | -0.519 | -0.033 | 0.313 | 0.160 | 0.206 |
|  |  | O_3_ (μg/m^3^) | -0.791 | -0.677 | -0.823 | 1 | 0.142 | -0.284 | 0.331 | 0.033 | -0.248 | -0.114 | -0.175 |
|  | **Built environment** | Distance water, m | -0.192 | -0.198 | -0.142 | 0.142 | 1 | -0.102 | 0.089 | -0.219 | -0.401 | 0.096 | 0.062 |
|  |  | Impervious surface,% | 0.390 | 0.335 | 0.411 | -0.284 | -0.102 | 1 | -0.696 | 0.019 | 0.208 | 0.067 | 0.079 |
|  |  | NDVI | -0.492 | -0.386 | -0.519 | 0.331 | 0.089 | -0.696 | 1 | 0.003 | -0.243 | -0.115 | -0.119 |
|  | **Temperature** | Mean T cool (^o^C) | -0.189 | -0.314 | -0.033 | 0.033 | -0.219 | 0.019 | 0.003 | 1 | 0.568 | -0.940 | -0.897 |
|  |  | Mean T warm (^o^C) | 0.234 | 0.187 | 0.313 | -0.248 | -0.401 | 0.208 | -0.243 | 0.568 | 1 | -0.282 | -0.151 |
|  |  | SD T cool (^o^C) | 0.305 | 0.423 | 0.160 | -0.114 | 0.096 | 0.067 | -0.115 | -0.940 | -0.282 | 1 | 0.970 |
|  |  | SD T warm (^o^C) | 0.355 | 0.479 | 0.206 | -0.175 | 0.062 | 0.079 | -0.119 | -0.897 | -0.151 | 0.970 | 1 |
|  |  | **Exposure** | **Air pollution** | | | | **Built environment** | | | **Temperature** | | | |
|  |  |  | NO_2_ (μg/m^3^) | PM_2.5_ (μg/m^3^) | BC  (10^5^/m) | O_3_ (μg/m^3^) | Distance water, m | Impervious surface, % | NDVI | Mean Ta cool (^o^C) | Mean Ta warm (^o^C) | SD Ta cool (^o^C) | SD Ta warm (^o^C) |
| **Sweden** | **Air pollution** | NO_2_ (μg/m^3^) | 1 | 0.732 | 0.870 | -0.170 | 0.004 | 0.581 | -0.684 | 0.384 | 0.386 | -0.258 | -0.159 |
|  |  | PM_2.5_ (μg/m^3^) | 0.732 | 1 | 0.780 | 0.311 | 0.094 | 0.376 | -0.422 | 0.727 | 0.577 | -0.464 | -0.487 |
|  |  | BC (10^-5^/m) | 0.870 | 0.780 | 1 | 0.070 | 0.078 | 0.465 | -0.554 | 0.659 | 0.624 | -0.492 | -0.303 |
|  |  | O_3_ (μg/m^3^) | -0.170 | 0.311 | 0.070 | 1 | 0.182 | -0.249 | 0.268 | 0.639 | 0.407 | -0.535 | -0.420 |
|  | **Built environment** | Distance water, m | 0.004 | 0.094 | 0.078 | 0.182 | 1 | -0.104 | 0.104 | 0.167 | 0.190 | -0.091 | -0.090 |
|  |  | Impervious surface,% | 0.581 | 0.376 | 0.465 | -0.249 | -0.104 | 1 | -0.736 | 0.111 | 0.138 | -0.069 | -0.012 |
|  |  | NDVI | -0.684 | -0.422 | -0.554 | 0.268 | 0.104 | -0.736 | 1 | -0.149 | -0.221 | 0.154 | -0.076 |
|  | **Temperature** | Mean T cool (^o^C) | 0.384 | 0.727 | 0.659 | 0.639 | 0.167 | 0.111 | -0.149 | 1 | 0.817 | -0.845 | -0.489 |
|  |  | Mean T warm (^o^C) | 0.386 | 0.577 | 0.624 | 0.407 | 0.190 | 0.138 | -0.221 | 0.817 | 1 | -0.643 | -0.244 |
|  |  | SD T cool (^o^C) | -0.258 | -0.464 | -0.492 | -0.535 | -0.091 | -0.069 | 0.154 | -0.845 | -0.643 | 1 | 0.121 |
|  |  | SD T warm (^o^C) | -0.159 | -0.487 | -0.303 | -0.420 | -0.090 | -0.012 | -0.076 | -0.489 | -0.244 | 0.121 | 1 |
|  |  | **Exposure** | **Air pollution** | | | | **Built environment** | | | **Temperature** | | | |
|  |  |  | NO_2_ (μg/m^3^) | PM_2.5_ (μg/m^3^) | BC  (10^5^/m) | O_3_ (μg/m^3^) | Distance water, m | Impervious surface, % | NDVI | Mean Ta cool (^o^C) | Mean Ta warm (^o^C) | SD Ta cool (^o^C) | SD Ta warm (^o^C) |
| **Swiss** | **Air pollution** | NO_2_ (μg/m^3^) | 1 | 0.702 | 0.929 | -0.664 | -0.250 | 0.533 | -0.658 | 0.524 | 0.539 | -0.368 | -0.130 |
|  |  | PM_2.5_ (μg/m^3^) | 0.702 | 1 | 0.695 | -0.599 | -0.128 | 0.354 | -0.425 | 0.680 | 0.697 | -0.475 | -0.182 |
|  |  | BC (10^-5^/m) | 0.929 | 0.695 | 1 | -0.674 | -0.262 | 0.489 | -0.607 | 0.495 | 0.510 | -0.330 | -0.120 |
|  |  | O_3_ (μg/m^3^) | -0.664 | -0.599 | -0.674 | 1 | 0.111 | -0.385 | 0.431 | -0.564 | -0.574 | 0.233 | -0.222 |
|  | **Built environment** | Distance water, m | -0.250 | -0.128 | -0.262 | 0.111 | 1 | -0.206 | 0.296 | -0.044 | -0.066 | -0.005 | -0.019 |
|  |  | Impervious surface,% | 0.533 | 0.354 | 0.489 | -0.385 | -0.206 | 1 | -0.687 | 0.166 | 0.173 | -0.070 | 0.052 |
|  |  | NDVI | -0.658 | -0.425 | -0.607 | 0.431 | 0.296 | -0.687 | 1 | -0.196 | -0.220 | 0.046 | -0.053 |
|  | **Temperature** | Mean T cool (^o^C) | 0.524 | 0.680 | 0.495 | -0.564 | -0.044 | 0.166 | -0.196 | 1 | 0.982 | -0.805 | -0.346 |
|  |  | Mean T warm (^o^C) | 0.539 | 0.697 | 0.510 | -0.574 | -0.066 | 0.173 | -0.220 | 0.982 | 1 | -0.733 | -0.341 |
|  |  | SD T cool (^o^C) | -0.368 | -0.475 | -0.330 | 0.233 | -0.005 | -0.070 | 0.046 | -0.805 | -0.733 | 1 | 0.601 |
|  |  | SD T warm (^o^C) | -0.130 | -0.182 | -0.120 | -0.222 | -0.019 | 0.052 | -0.053 | -0.346 | -0.341 | 0.601 | 1 |

**B. Traditioanl adult cohorts**

|  |  | **Exposure** | **Air pollution** | | | | **Built environment** | | | **Temperature** | | | |
| --- | --- | --- | --- | --- | --- | --- | --- | --- | --- | --- | --- | --- | --- |
| **Cohort** |  |  | NO_2_ (μg/m^3^) | PM_2.5_ (μg/m^3^) | BC  (10^5^/m) | O_3_ (μg/m^3^) | Distance water, m | Impervious surface, % | NDVI | Mean Ta cool (^o^C) | Mean Ta warm (^o^C) | SD Ta cool (^o^C) | SD Ta warm (^o^C) |
| **CEANS** | **Air pollution** | NO_2_ (μg/m^3^) | 1 | 0.698 | 0.840 | -0.714 | -0.123 | 0.622 | -0.788 | -0.068 | 0.240 | 0.122 | -0.238 |
|  |  | PM_2.5_ (μg/m^3^) | 0.698 | 1 | 0.601 | -0.418 | -0.146 | 0.378 | -0.475 | 0.134 | 0.060 | -0.064 | -0.289 |
|  |  | BC (10^-5^/m) | 0.840 | 0.601 | 1 | -0.640 | -0.186 | 0.467 | -0.648 | 0.150 | 0.058 | -0.084 | -0.452 |
|  |  | O_3_ (μg/m^3^) | -0.714 | -0.418 | -0.640 | 1 | -0.058 | -0.407 | 0.516 | 0.337 | -0.365 | -0.339 | -0.107 |
|  | **Built environment** | Distance water, m | -0.123 | -0.146 | -0.186 | -0.058 | 1 | -0.116 | 0.151 | -0.361 | 0.260 | 0.331 | 0.359 |
|  |  | Impervious surface,% | 0.622 | 0.378 | 0.467 | -0.407 | -0.116 | 1 | -0.735 | -0.088 | 0.173 | 0.117 | -0.106 |
|  |  | NDVI | -0.788 | -0.475 | -0.648 | 0.516 | 0.151 | -0.735 | 1 | 0.079 | -0.214 | -0.125 | 0.198 |
|  | **Temperature** | Mean T cool (^o^C) | -0.068 | 0.134 | 0.150 | 0.337 | -0.361 | -0.088 | 0.079 | 1 | -0.922 | -0.986 | -0.853 |
|  |  | Mean T warm (^o^C) | 0.240 | 0.060 | 0.058 | -0.365 | 0.260 | 0.173 | -0.214 | -0.922 | 1 | 0.968 | 0.717 |
|  |  | SD T cool (^o^C) | 0.122 | -0.064 | -0.084 | -0.339 | 0.331 | 0.117 | -0.125 | -0.986 | 0.968 | 1 | 0.819 |
|  |  | SD T warm (^o^C) | -0.238 | -0.289 | -0.452 | -0.107 | 0.359 | -0.106 | 0.198 | -0.853 | 0.717 | 0.819 | 1 |
|  |  | **Exposure** | **Air pollution** | | | | **Built environment** | | | **Temperature** | | | |
|  |  |  | NO_2_ (μg/m^3^) | PM_2.5_ (μg/m^3^) | BC  (10^5^/m) | O_3_ (μg/m^3^) | Distance water, m | Impervious surface, % | NDVI | Mean Ta cool (^o^C) | Mean Ta warm (^o^C) | SD Ta cool (^o^C) | SD Ta warm (^o^C) |
| **EPIC-NL** | **Air pollution** | NO_2_ (μg/m^3^) | 1 | 0.233 | 0.837 | -0.739 | -0.122 | 0.335 | -0.578 | 0.498 | -0.543 | -0.481 | -0.525 |
|  |  | PM_2.5_ (μg/m^3^) | 0.233 | 1 | 0.420 | 0.056 | -0.208 | 0.288 | -0.370 | -0.219 | 0.298 | 0.400 | 0.348 |
|  |  | BC (10^-5^/m) | 0.837 | 0.420 | 1 | -0.549 | -0.110 | 0.308 | -0.570 | 0.273 | -0.305 | -0.176 | -0.245 |
|  |  | O_3_ (μg/m^3^) | -0.739 | 0.056 | -0.549 | 1 | 0.113 | -0.183 | 0.395 | -0.763 | 0.718 | 0.802 | 0.779 |
|  | **Built environment** | Distance water, m | -0.122 | -0.208 | -0.110 | 0.113 | 1 | 0.231 | -0.172 | -0.082 | -0.036 | 0.053 | 0.023 |
|  |  | Impervious surface,% | 0.335 | 0.288 | 0.308 | -0.183 | -0.172 | 1 | -0.606 | 0.092 | -0.075 | -0.051 | -0.067 |
|  |  | NDVI | -0.578 | -0.370 | -0.570 | 0.395 | 0.231 | -0.606 | 1 | -0.245 | 0.244 | 0.170 | 0.217 |
|  | **Temperature** | Mean T cool (^o^C) | 0.498 | -0.219 | 0.273 | -0.763 | -0.082 | 0.092 | -0.245 | 1 | -0.835 | -0.924 | -0.943 |
|  |  | Mean T warm (^o^C) | -0.543 | 0.298 | -0.305 | 0.718 | -0.036 | -0.075 | 0.244 | -0.835 | 1 | 0.864 | 0.951 |
|  |  | SD T cool (^o^C) | -0.481 | 0.400 | -0.176 | 0.802 | 0.053 | -0.051 | 0.170 | -0.924 | 0.864 | 1 | 0.964 |
|  |  | SD T warm (^o^C) | -0.525 | 0.348 | -0.245 | 0.779 | 0.023 | -0.067 | 0.217 | -0.943 | 0.951 | 0.964 | 1 |
|  |  | **Exposure** | **Air pollution** | | | | **Built environment** | | | **Temperature** | | | |
|  |  |  | NO_2_ (μg/m^3^) | PM_2.5_ (μg/m^3^) | BC  (10^5^/m) | O_3_ (μg/m^3^) | Distance water, m | Impervious surface, % | NDVI, units | Mean Ta cool (^o^C) | Mean Ta warm (^o^C) | SD Ta cool (^o^C) | SD Ta warm (^o^C) |
| **KORA** | **Air pollution** | NO_2_ (μg/m^3^) | 1 | 0.653 | 0.924 | -0.898 | -0.403 | 0.514 | -0.679 | -0.132 | -0.143 | -0.330 | -0.432 |
|  |  | PM_2.5_ (μg/m^3^) | 0.653 | 1 | 0.584 | -0.544 | -0.270 | 0.443 | -0.462 | -0.117 | -0.080 | 0.007 | -0.178 |
|  |  | BC (10^-5^/m) | 0.924 | 0.584 | 1 | -0.904 | -0.414 | 0.452 | -0.640 | -0.276 | -0.286 | -0.342 | -0.548 |
|  |  | O_3_ (μg/m^3^) | -0.898 | -0.544 | -0.904 | 1 | 0.302 | -0.400 | 0.558 | 0.121 | 0.135 | 0.324 | 0.411 |
|  | **Built environment** | Distance water, m | -0.403 | -0.270 | -0.414 | 0.302 | 1 | -0.294 | 0.463 | 0.208 | 0.195 | 0.163 | 0.254 |
|  |  | Impervious surface,% | 0.514 | 0.443 | 0.452 | -0.400 | -0.294 | 1 | -0.621 | -0.184 | -0.154 | -0.066 | -0.249 |
|  |  | NDVI | -0.679 | -0.462 | -0.640 | 0.558 | 0.463 | -0.621 | 1 | 0.197 | 0.152 | 0.092 | 0.301 |
|  | **Temperature** | Mean T cool (^o^C) | -0.132 | -0.117 | -0.276 | 0.121 | 0.208 | -0.184 | 0.197 | 1 | 0.952 | -0.010 | 0.744 |
|  |  | Mean T warm (^o^C) | -0.143 | -0.080 | -0.286 | 0.135 | 0.195 | -0.154 | 0.152 | 0.952 | 1 | 0.259 | 0.839 |
|  |  | SD T cool (^o^C) | -0.330 | 0.007 | -0.342 | 0.324 | 0.163 | -0.066 | 0.092 | -0.010 | 0.259 | 1 | 0.581 |
|  |  | SD T warm (^o^C) | -0.432 | -0.178 | -0.548 | 0.411 | 0.254 | -0.249 | 0.301 | 0.744 | 0.839 | 0.581 | 1 |

Abbreviations: BC, Black carbon; CEANS, Cardiovascular Effects of Air Pollution and Noise in Stockholm study; EPIC-NL, The European Prospective Investigation into Cancer and Nutrition- Netherlands; KORA, Cooperative Health Research in the Augsburg Region; NDVI, Normalized Difference Vegetation Index; NO_2_, nitrogen dioxide; O_3_, ozone during warm months (April to September); PM_2.5_, particulate matter with an aerodynamic diameter of less than 2.5 μm; m: meters; T, Temperature; SD, Standard deviation; warm: months from April to September; cool: months from January to March & October to December).

**Table S3.** Hazard Ratios (HR) and 95% Confidence Intervals (95% CI) from single-exposure Cox models for all-cause mortality.

1. **Administrative Cohorts**

|  | **HR (95% C.I.)** | | | | | | | | | | |
| --- | --- | --- | --- | --- | --- | --- | --- | --- | --- | --- | --- |
|  | **Air pollution** | | | | **Built environment** | | | **Temperature** | | | |
| **Administrative**  **Cohort Name** | NO_2_  *per 10 μg/m^3^* | PM_2.5_  *per 5 μg/m^3^* | BC  *per 0.5 10^-5^/m^-1^* | O_3_  *per 10 μg/m^3^* | Distance water  *per 1000 m* | Impervious surface  *per 10 % un.* | NDVI  *per 0.1 units* | Mean T cool  *per 1.0 ^o^C* | Mean T warm  *per 1.0 ^o^C* | SD T cool *per 1.0 ^o^C* | SD T warm  *per 1.0 ^o^C* |
| **Model 1** | | | | | | | | | | | |
| **Catalonia** | 1.015 (1.005-1.024) | 1.073 (1.046-1.100) | 0.992 (0.981-1.003) | 0.988 (0.973-1.004) | 0.988 (0.985-0.991) | 0.999 (0.997-1.001) | 1.010 (1.004-1.015) | 1.028 (1.019-1.037) | 1.047 (1.033-1.061) | 0.883 (0.830-0.941) | 0.844 (0.787-0.906) |
| **Greece** | 0.996 (0.994-0.999) | 1.034 (1.029-1.039) | 0.992 (0.988-0.995) | 1.003 (0.999-1.007) | 0.997 (0.996-0.998) | 1.003 (1.002-1.004) | 0.996 (0.994-0.998) | 1.004 (1.002-1.006) | 1.018 (1.016-1.021) | 1.035 (1.027-1.044) | 1.026 (1.016-1.036) |
| **Netherlands** | 1.129 (1.125-1.133) | 1.194 (1.185-1.203) | 1.133 (1.129-1.137) | 0.883 (0.88-0.887) | 0.980 (0.978-0.981) | 1.025 (1.024-1.026) | 0.933 (0.931-0.935) | 1.011 (1.004-1.017) | 1.022 (1.012-1.032) | 1.016 (1.008-1.025) | 0.997 (0.988-1.007) |
| **Rome** | 0.971 (0.964-0.978) | 0.961 (0.937-0.985) | 0.973 (0.966-0.981) | 1.067 (1.051-1.082) | 1.003 (1.001-1.005) | 1.003 (1.001-1.005) | 0.994 (0.990-0.998) | 0.961 (0.952-0.97) | 0.958 (0.936-0.98) | 1.177 (1.137-1.219) | 1.137 (1.098-1.178) |
| **Sweden** | 1.028 (1.024-1.031) | 1.036 (1.03-1.043) | 1.011 (1.007-1.015) | 0.955 (0.948-0.961) | 1.003 (1.001-1.006) | 1.013 (1.012-1.014) | 0.974 (0.972-0.976) | 0.992 (0.99-0.994) | 0.986 (0.983-0.99) | 1.016 (1.01-1.022) | 1.007 (0.995-1.019) |
| **Switzerland** | 1.024 (1.019-1.029) | 0.991 (0.984-0.997) | 1.022 (1.017-1.027) | 0.972 (0.966-0.978) | 1.002 (1.001-1.004) | 1.021 (1.019-1.022) | 0.965 (0.963-0.968) | 0.991 (0.99-0.992) | 0.990 (0.988-0.991) | 1.059 (1.049-1.069) | 1.130 (1.099-1.162) |
| **Pooled** | 1.026 (0.972 – 1.083) | 1.046 (0.966 - 1.132) | 1.019 (0.963 -1.079 | 0.976 (0.915 – 1.042) | 0.996 (0.985 – 1.006) | 1.011 (0.999 – 1.022) | 0.978 (0.949 – 1.008) | 0.998 (0.974 – 1.021) | 1.004 (0.972 – 1.037) | 1.030 (0.937 – 1.131) | 1.021 (0.914 – 1.141( |
| **Heterogeneity I^2^** | 100% | 100% | 100% | 100% | 99% | 100% | 100% | 98% | 99% | 96% | 97% |
| **Model 2** | | | | | | | | | | | |
| **Catalonia** | 1.019 (1.009-1.028) | 1.079 (1.052-1.106) | 1.015 (1.004-1.027) | 0.971 (0.956-0.986) | 0.989 (0.985-0.992) | 0.997 (0.995-0.999) | 1.010 (1.005-1.016) | 1.026 (1.018-1.035) | 1.040 (1.027-1.054) | 0.884 (0.831-0.942) | 0.839 (0.782-0.899) |
| **Greece** | 0.996 (0.994-0.999) | 1.034 (1.029-1.039) | 0.992 (0.988-0.995) | 1.003 (0.999-1.007) | 0.997 (0.996-0.998) | 1.003 (1.002-1.004) | 0.996 (0.994-0.998) | 1.004 (1.002-1.006) | 1.018 (1.016-1.021) | 1.035 (1.027-1.044) | 1.026 (1.016-1.036) |
| **Netherlands** | 1.044 (1.040-1.047) | 1.049 (1.041-1.058) | 1.053 (1.049-1.056) | 0.949 (0.946-0.953) | 0.995 (0.993-0.997) | 1.006 (1.005-1.007) | 0.985 (0.983-0.987) | 1.008 (1.002-1.015) | 1.023 (1.014-1.033) | 1.012 (1.003-1.02) | 1.005 (0.995-1.014) |
| **Rome** | 0.989 (0.982-0.996) | 1.009 (0.984-1.034) | 0.987 (0.98-0.995) | 1.030 (1.016-1.045) | 0.996 (0.993-0.998) | 0.999 (0.997-1.001) | 1.000 (0.996-1.003) | 0.981 (0.972-0.99) | 0.994 (0.972-1.017) | 1.093 (1.056-1.132) | 1.077 (1.040-1.116) |
| **Sweden** | 1.028 (1.024-1.031) | 1.036 (1.03-1.043) | 1.011 (1.007-1.015) | 0.955 (0.948-0.961) | 1.003 (1.001-1.006) | 1.013 (1.012-1.014) | 0.974 (0.972-0.976) | 0.992 (0.99-0.994) | 0.986 (0.983-0.99) | 1.016 (1.01-1.022) | 1.007 (0.995-1.019) |
| **Switzerland** | 1.025 (1.020-1.030) | 1.011 (1.004-1.018) | 1.024 (1.019-1.029) | 0.970 (0.964-0.976) | 0.997 (0.995-0.998) | 1.015 (1.014-1.016) | 0.977 (0.974-0.980) | 0.998 (0.997-1.000) | 0.998 (0.996-1.000) | 1.008 (0.998-1.018) | 1.006 (0.977-1.036) |
| **Pooled** | 1.017 (0.995 – 1.038) | 1.035 (1.010 – 1.061) | 1.014 (0.989 – 1.039) | 0.979 (0.947 – 1.012) | 0.996 (0.991 – 1.001) | 1.005 (0.998 – 1.013) | 0.990 (0.975 – 1.005) | 1.002 (0.986 – 1.017) | 1.010 (0.989 – 1.032) | 1.010 (0.943 – 1.082) | 0.995 (0.914 – 1.083) |
| **Heterogeneity I^2^** | 99% | 93% | 99% | 99% | 91% | 99% | 99% | 96% | 99% | 92% | 90% |
| **Model 3** | | | | | | | | | | | |
| **Catalonia** | 1.028 (1.019-1.038) | 1.099 (1.072-1.127) | 1.031 (1.019-1.042) | 0.951 (0.935-0.966) | 0.992 (0.988-0.995) | 0.997 (0.995-1.000) | 1.009 (1.003-1.015) | 1.030 (1.022-1.039) | 1.045 (1.031-1.059) | 0.856 (0.804-0.912) | 0.823 (0.768-0.883) |
| **Greece** | 1.012 (1.009-1.016) | 1.047 (1.041-1.053) | 1.011 (1.007-1.016) | 1.001 (0.997-1.005) | 0.997 (0.996-0.998) | 1.005 (1.004-1.006) | 0.989 (0.987-0.991) | 1.004 (1.002-1.006) | 1.020 (1.018-1.022) | 1.031 (1.022-1.039) | 1.025 (1.016-1.035) |
| **Netherlands** | 1.027 (1.023-1.031) | 1.014 (1.006-1.022) | 1.038 (1.034-1.042) | 0.964 (0.96-0.968) | 0.999 (0.997-1.001) | 1.003 (1.002-1.004) | 0.999 (0.997-1.001) | 1.006 (1.000-1.013) | 1.017 (1.007-1.026) | 1.010 (1.002-1.019) | 1.002 (0.993-1.011) |
| **Rome** | 1.016 (1.008-1.025) | 1.058 (1.029-1.088) | 1.014 (1.005-1.023) | 0.988 (0.973-1.004) | 0.994 (0.991-0.997) | 1.003 (1.001-1.005) | 0.991 (0.986-0.995) | 0.996 (0.987-1.006) | 1.029 (1.005-1.054) | 1.040 (1.004-1.077) | 1.034 (0.998-1.072) |
| **Sweden** | 1.076 (1.072-1.08) | 1.095 (1.088-1.102) | 1.055 (1.051-1.06) | 0.941 (0.935-0.948) | 0.994 (0.991-0.997) | 1.018 (1.017-1.019) | 0.958 (0.956-0.96) | 1.001 (0.999-1.003) | 0.993 (0.990-0.997) | 1.000 (0.994-1.005) | 1.008 (0.996-1.020) |
| **Switzerland** | 1.040 (1.034-1.046) | 1.019 (1.01-1.027) | 1.044 (1.037-1.051) | 0.953 (0.945-0.961) | 0.999 (0.997-1) | 1.016 (1.015-1.017) | 0.973 (0.969-0.976) | 0.999 (0.998-1.001) | 0.999 (0.997-1.001) | 1.000 (0.989-1.011) | 0.983 (0.953-1.013) |
| **Pooled** | 1.033 (1.009 – 1.058) | 1.054 (1.016 – 1.093) | 1.032 (1.014 – 1.050) | 0.966 (0.942 – 0.991) | 0.996 (0.993 – 0.999) | 1.007 (0.998 – 1.016) | 0.986 (0.967 – 1.006) | 1.006 (0.993 – 1.018) | 1.016 (0.996 – 1.036) | 0.992 (0.926 – 1.063) | 0.981 (0.902 – 1.068) |
| **Heterogeneity I^2^** | 99% | 98% | 98% | 98% | 91% | 99% | 99% | 91% | 98% | 93% | 90% |

**B. Traditional adult cohorts**

|  | **HR (95% C.I.)** | | | | | | | | | | |
| --- | --- | --- | --- | --- | --- | --- | --- | --- | --- | --- | --- |
|  | **Air pollution** | | | | **Built environment** | | | **Temperature** | | | |
| **Adult Cohort Name** | NO_2_  *per 10 μg/m^3^* | PM_2.5_  *per 5 μg/m^3^* | BC  *per 0.5 10^-5^/m^-1^* | O_3_  *per 10 μg/m^3^* | Distance water  *per 1000 m* | Impervious surface  *per 10 % un.* | NDVI  *per 0.1 units* | Mean Ta cool  *per 1.0 ^o^C* | Mean Ta warm  *per 1.0 ^o^C* | SD Ta cool *per 1.0 ^o^C* | SD Ta warm  *per 1.0 ^o^C* |
| **Model 1** | | | | | | | | | | | |
| **CEANS** | 1.055 (0.988-1.127) | 1.132 (0.908-1.412) | 1.065 (0.981-1.158) | 0.905 (0.784-1.045) | 1.038 (0.984-1.095) | 1.028 (1.013-1.044) | 0.940 (0.904-0.977) | 1.005 (0.913-1.106) | 0.940 (0.780-1.132) | 0.982 (0.879-1.097) | 0.971 (0.500-1.886) |
| **EPIC-NL** | 1.237 (1.102-1.389) | 0.947 (0.661-1.357) | 1.128 (1.003-1.269) | 0.909 (0.798-1.035) | 1.025 (0.951-1.105) | 1.023 (0.995-1.052) | 0.927 (0.863-0.995) | 1.097 (0.731-1.646) | 0.923 (0.651-1.309) | 0.807 (0.512-1.271) | 0.878 (0.653-1.181) |
| KORA | 1.036 (0.943-1.137) | 1.151 (0.847-1.563) | 1.034 (0.955-1.121) | 0.913 (0.761-1.095) | 0.979 (0.955-1.004) | 1.014 (0.991-1.038) | 0.94 (0.893-0.989) | 0.848 (0.536-1.341) | 0.878 (0.598-1.289) | 0.616 (0.166-2.286) | 0.397 (0.089-1.783) |
| **Pooled** | 1.098 (0.871 – 1.384) | 1.098 (0.877 – 1.374) | 1.064 (0.966 – 1.172) | 0.908 (0.900 – 0.917) | 1.006 (0.927 – 1.091) | 1.024 (1.006 – 1.042) | 0.938 (0.923 – 0.953) | 1.002 (0.890 – 1.129) | 0.927 (0.861 – 0.997) | 0.968 (0.811 – 1.157) | 0.870 (0.558 – 1.357) |
| **Heterogeneity I^2^** | 69% | 0% | 0% | 0% | 55% | 0% | 0% | 0% | 0% | 0% | 0% |
| **Model 2** | | | | | | | | | | | |
| **CEANS** | 1.024 (0.958-1.094) | 1.089 (0.874-1.358) | 1.037 (0.954-1.128) | 0.933 (0.808-1.077) | 1.032 (0.978-1.089) | 1.016 (1.000-1.032) | 0.970 (0.932-1.009) | 1.000 (0.908-1.101) | 0.953 (0.791-1.150) | 0.990 (0.885-1.106) | 1.048 (0.537-2.044) |
| **EPIC-NL** | 1.052 (0.931-1.189) | 1.123 (0.754-1.671) | 0.994 (0.88-1.123) | 1.048 (0.909-1.208) | 1.027 (0.949-1.112) | 0.992 (0.965-1.02) | 1.007 (0.936-1.083) | 0.766 (0.484-1.212) | 1.419 (0.947-2.127) | 1.174 (0.703-1.962) | 1.242 (0.877-1.758) |
| **KORA** | 0.933 (0.847-1.027) | 0.931 (0.685-1.266) | 0.947 (0.872-1.028) | 1.11 (0.92-1.339) | 0.993 (0.968-1.018) | 0.997 (0.974-1.02) | 0.988 (0.938-1.04) | 0.948 (0.593-1.516) | 0.945 (0.637-1.401) | 0.901 (0.241-3.372) | 0.876 (0.189-4.047) |
| **Pooled** | 1.001 (0.864 – 1.159) | 1.047 (0.834 – 1.315) | 0.991 (0.876 – 1.121) | 1.017 (0.820 – 1.261) | 1.006 (0.953 – 1.062) | 1.005 (0.972 – 1.038) | 0.981 (0.941 – 1023) | 0.987 (0.840 – 1.160) | 1.039 (0.626 – 1.724) | 0.996 (0.982 – 1.113) | 1.183 (0.896 – 1.561) |
| **Heterogeneity I^2^** | 37% | 0% | 14% | 16% | 3% | 36% | 0% | 0% | 37% | 0% | 0% |
| **Model 3** | | | | | | | | | | | |
| **CEANS** | 1.026 (0.961-1.096) | 1.084 (0.871-1.351) | 1.053 (0.968-1.145) | 0.905 (0.784-1.045) | 1.026 (0.972-1.083) | 1.014 (0.999-1.03) | 0.976 (0.937-1.016) | 1.013 (0.92-1.115) | 0.926 (0.767-1.117) | 0.969 (0.867-1.084) | 0.983 (0.504-1.917) |
| **EPIC-NL** | 1.044 (0.915-1.191) | 1.113 (0.749-1.654) | 0.979 (0.859-1.116) | 1.049 (0.901-1.22) | 1.021 (0.942-1.107) | 0.988 (0.961-1.016) | 1.017 (0.941-1.099) | 0.839 (0.526-1.338) | 1.277 (0.844-1.933) | 1.032 (0.61-1.744) | 1.134 (0.794-1.620) |
| **KORA** | 0.889 (0.784-1.009) | 0.920 (0.659-1.285) | 0.895 (0.794-1.007) | 1.169 (0.928-1.472) | 0.991 (0.963-1.019) | 0.997 (0.973-1.021) | 0.981 (0.920-1.045) | 0.940 (0.571-1.547) | 0.938 (0.619-1.42) | 0.890 (0.226-3.512) | 0.817 (0.131-5.110) |
| **Pooled** | 0.991 (0.808 – 1.216) | 1.046 (0.835 – 1.311) | 0.980 (0.796 – 1.207) | 1.016 (0.747 – 1.381) | 1.001 (0.954 – 1.051) | 1.003 (0.970 – 1.037) | 0.983 (0.940 – 1.029) | 1.003 (0.891 – 1.128) | 0.972 (0.690 – 1.370) | 0.971 (0.930 – 1.015) | 1.089 (0.863 – 1.375) |
| **Heterogeneity I^2^** | 54% | 0% | 59% | 50% | 0% | 36% | 0% | 0% | 0% | 0% | 0% |

Abbreviations: BC, Black carbon; CEANS, Cardiovascular Effects of Air Pollution and Noise in Stockholm study; EPIC-NL, The European Prospective Investigation into Cancer and Nutrition- Netherlands; KORA, Cooperative Health Research in the Augsburg Region; NDVI, Normalized Difference Vegetation Index; NO_2_, nitrogen dioxide; O_3_, ozone during warm months (April to September); PM_2.5_, particulate matter with an aerodynamic diameter of less than 2.5 μm; m: meters; T, Temperature; SD, Standard deviation; warm: months from April to September; cool: months from January to March & October to December).

HRs are expressed per fixed increment.

Catalonia: HRs adjusted for age (time scale), sex (strata), smoking status, individual income, psca index, percentage of non-Spanish residents in census tract, and population density per m^2^.

Greece: HRs adjusted for age (time scale), sex (strata), NUTS1 areas country-wide (4 levels: Attica / Aegean Islands, Crete / North Greece / Central Greece)) & 4 area-level variables: tertiary education rate, unemployment rate, degree of urbanicity in 3 categories: 1. Cities (densely populated areas), 2. Towns and Suburbs (intermediate density areas) and 3. Rural areas (thinly populated areas) and married rate. For the Greater Area of Athens and other large municipalities (population greater than 100,000 inhabitants) in Greece, the aforementioned variables were available at square-block level. For the rest of the areas in Greece, the variables were available at municipality unit level.

Rome: HRs adjusted for age (timescale), sex (strata), place of birth, education level, employment status, marital status, citizenship, deprivation index on a census block level and unemployment rate, percentage of graduates and house prices on a neighborhood level.

Sweden: HRs adjusted for age (time scale), sex (strata), living condition, education level, district mean income, portion of people with high school or higher education in district, area.

Switzerland: HRs adjusted for age (time scale), strata(sex), Swiss region (n=7), marital status, occupational status, origin (i.e. Swiss vs. other), language region, socio-economic position index (SEP), community-level SEP index and community-level unemployment rate.

The Netherlands: HRs adjusted for age (time scale), sex (strata), area, wealth at 2010, categorized in deciles, partner status at 2010, individual socioeconomical status, area-level socio-economic status, area-level mean income at 2010, percentage of low-income households, urbanicity.

CEANS: HRs adjusted for subcohort (strata), age (timescale), sex (strata), and year of baseline visit, marital status, body-mass index, smoking (status, duration, intensity, intensity squared), employment status, education, and area-level socioeconomic status (2001 mean income on a neighborhood level).

EPIC-NL: HRs adjusted for subcohort (strata), age (timescale), sex (strata), and year of baseline visit, marital status, body-mass index, smoking (status, duration, intensity, intensity squared), employment status, education, and area-level socioeconomic status (2001 mean income on a neighborhood level).

KORA: HRs adjusted for subcohort (strata), age (timescale), sex (strata), and year of baseline visit, marital status, body-mass index, smoking (status, duration, intensity, intensity squared), employment status, education, and area-level socioeconomic status (Percentage of households with low income per 5 km² grid cell in 2007).

**Figures S1 to S22.** Forest plots for the cohort-specific and pooled associations between all-cause mortality and single exposures. Hazard Ratios (HR) and 95% Confidence Intervals (95% CI) from single-exposure Cox main model 3.

***S1.***


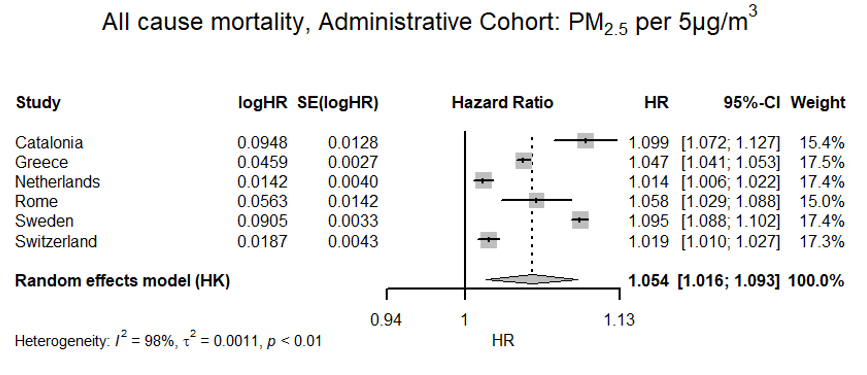


***S2.***

***
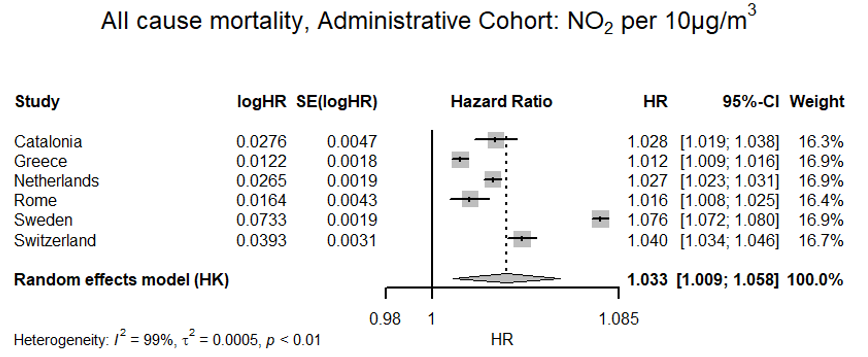
***

***S3.***

***
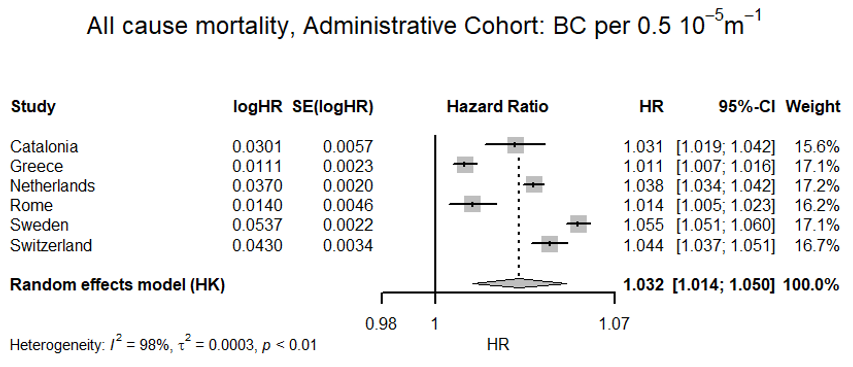
***

***S4.***


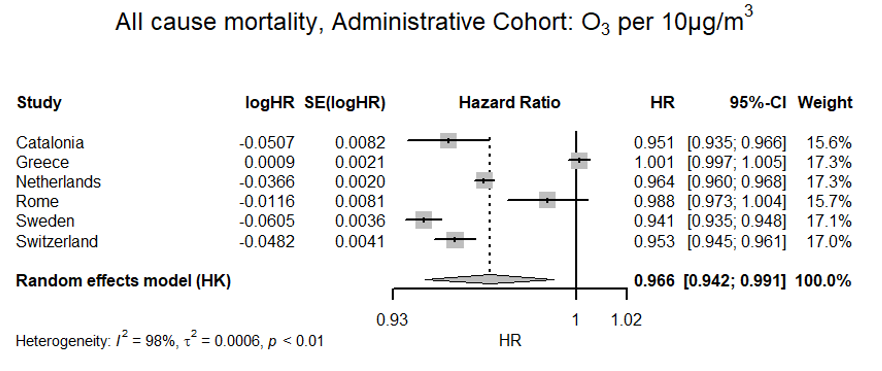


***S5.***


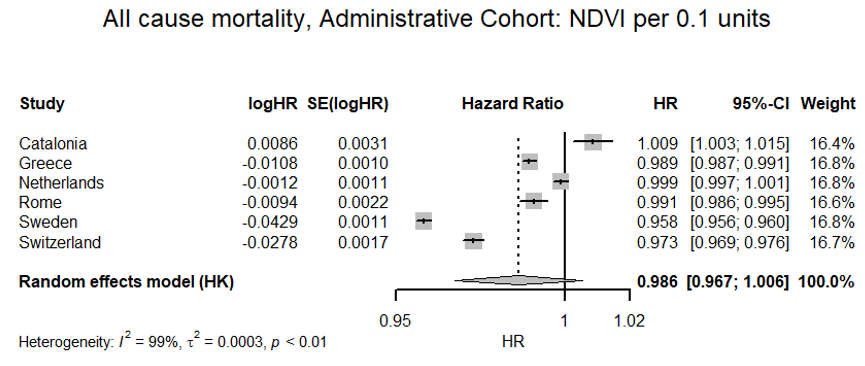


***S6.***


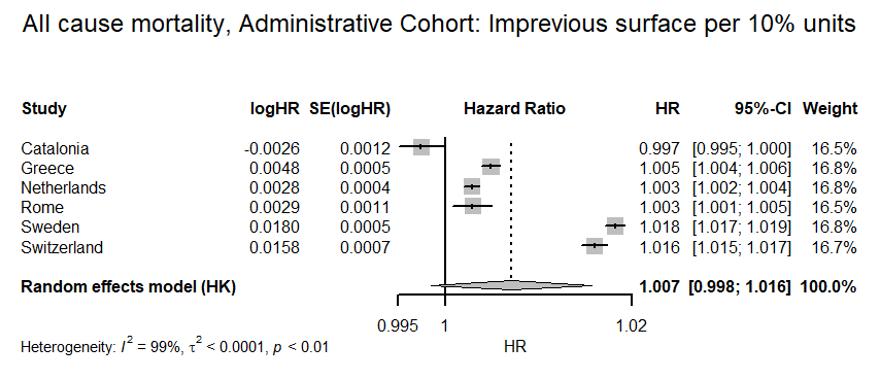


***S7.***


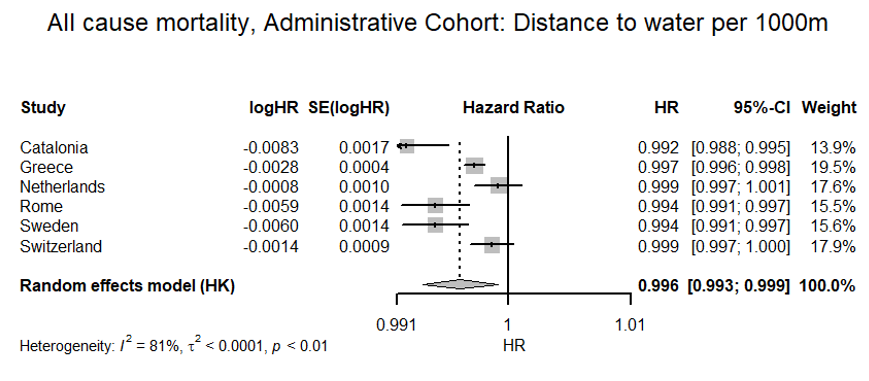


***S8.***


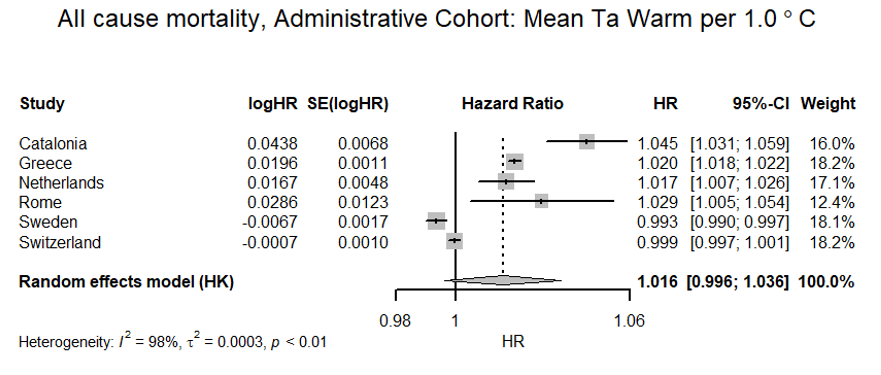


***S9.***


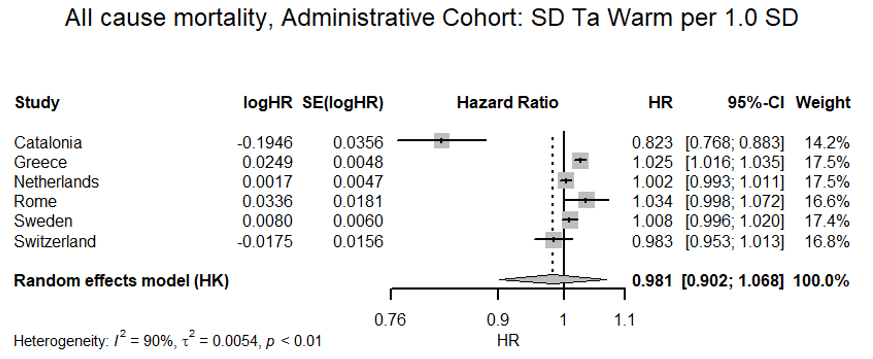


***S10.***


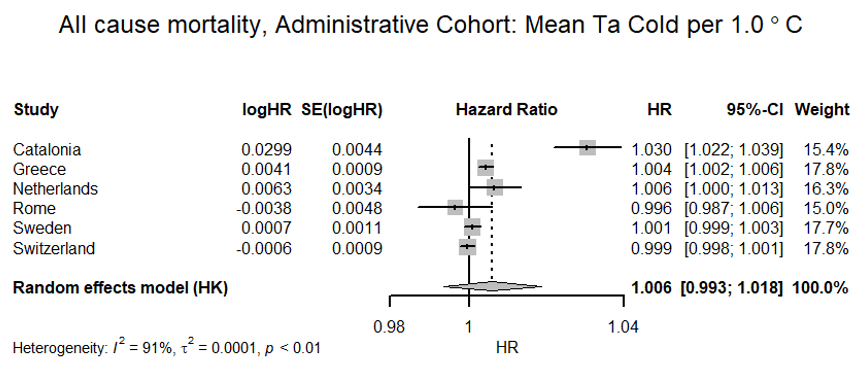


***S11.***


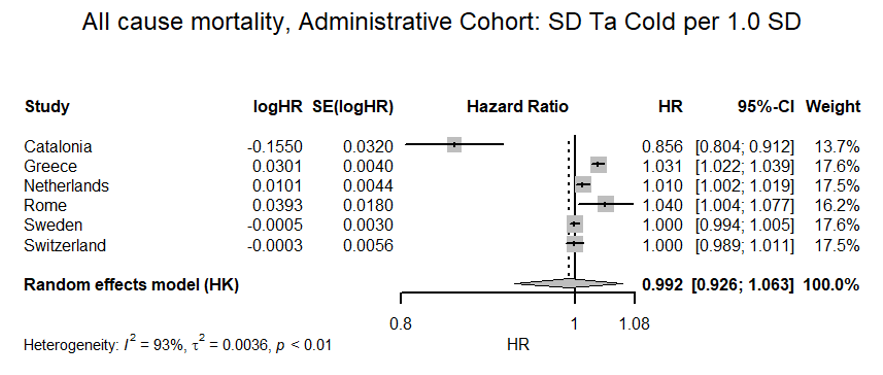


***S12.***


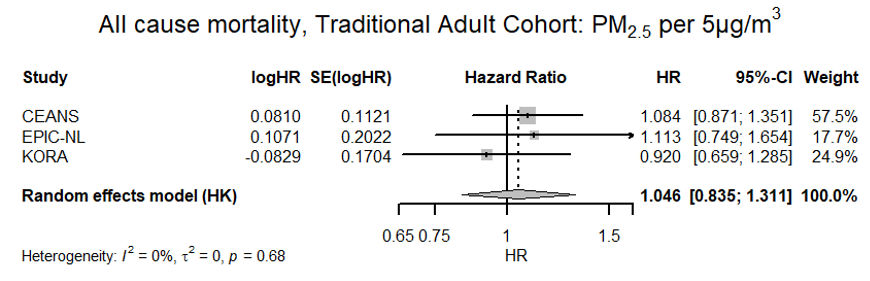


***S13.***


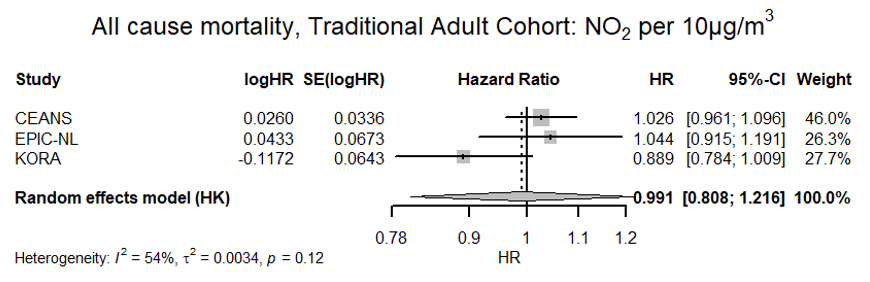


***S14.***


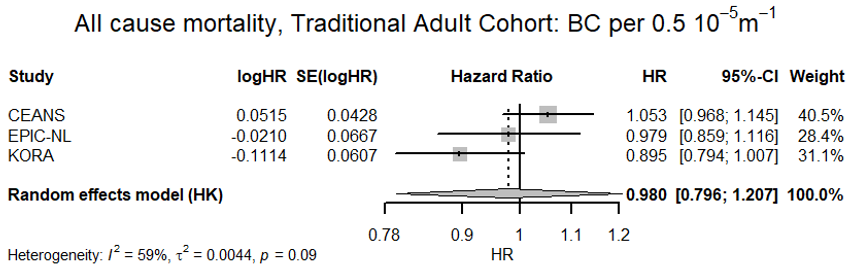


***S15.***

***
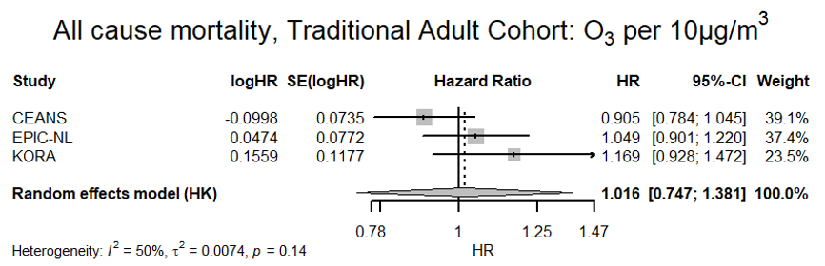
***

***S16.***

***
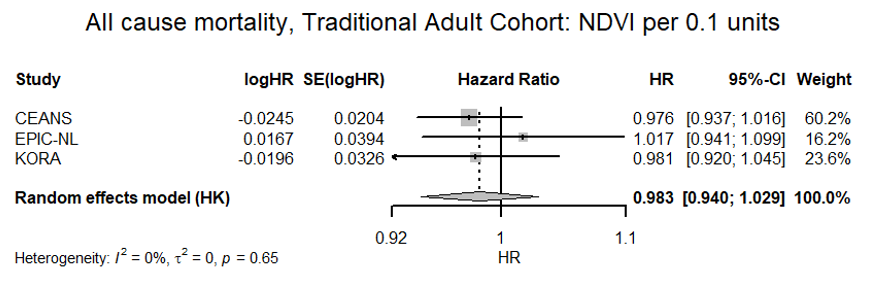
***

***S17.***

***
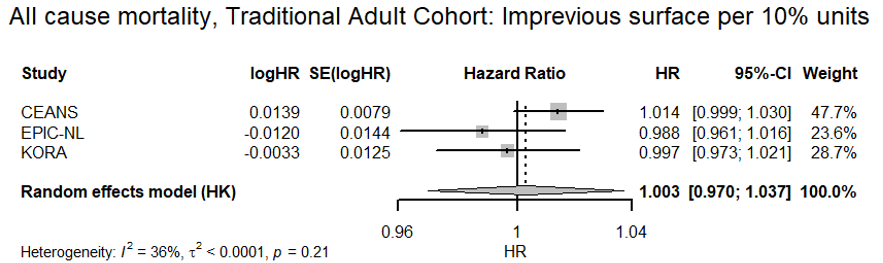
***

***S18.***

***
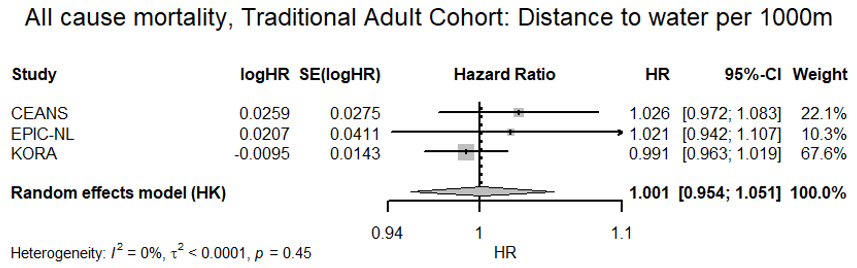
***

***S19.***

***
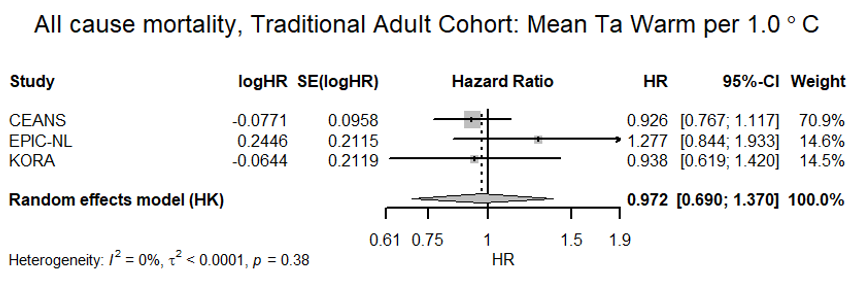
***

***S20.***

***
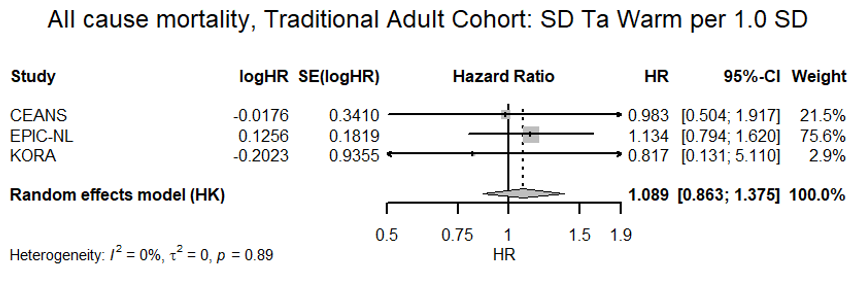
***

***S21.***

***
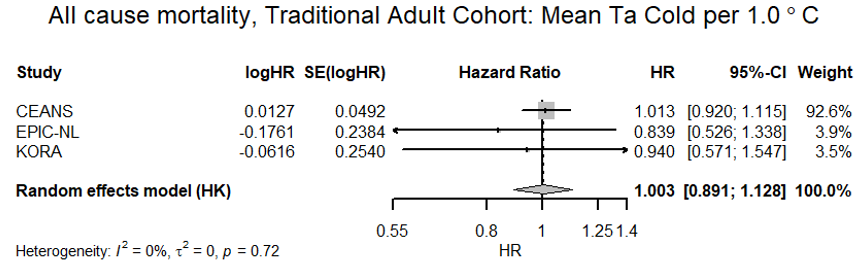
***

***S22.***

***
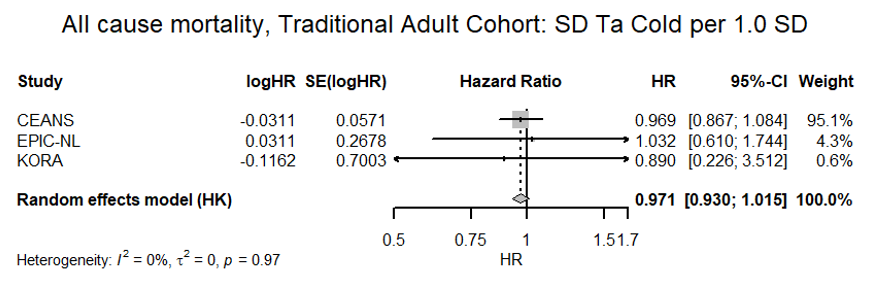
***

HRs are expressed per fixed increment.

Catalonia: HRs adjusted for age (time scale), sex (strata), smoking status, individual income, psca index, percentage of non-Spanish residents in census tract, and population density per m2.

Greece: HRs adjusted for age (time scale), sex (strata), NUTS1 areas country-wide (4 levels: Attica / Aegean Islands, Crete / North Greece / Central Greece)) & 4 area-level variables: tertiary education rate, unemployment rate, degree of urbanicity in 3 categories: 1. Cities (densely populated areas), 2. Towns and Suburbs (intermediate density areas) and 3. Rural areas (thinly populated areas) and married rate. For the Greater Area of Athens and other large municipalities (population greater than 100,000 inhabitants) in Greece, the aforementioned variables were available at square-block level. For the rest of the areas in Greece, the variables were available at municipality unit level.

Rome: HRs adjusted for age (timescale), sex (strata), place of birth, education level, employment status, marital status, citizenship, deprivation index on a census block level and unemployment rate, percentage of graduates and house prices on a neighborhood level.

Sweden: HRs adjusted for age (time scale), sex (strata), living condition, education level, district mean income, portion of people with high school or higher education in district, area.

Switzerland: HRs adjusted for age (time scale), strata(sex), Swiss region (n=7), marital status, occupational status, origin (i.e. Swiss vs. other), language region, socio-economic position index (SEP), community-level SEP index and community-level unemployment rate.

The Netherlands: HRs adjusted for age (time scale), sex (strata), area, wealth at 2010, categorized in deciles, partner status at 2010, individual socioeconomical status, area-level socio-economic status, area-level mean income at 2010, percentage of low-income households, urbanicity.

CEANS: HRs adjusted for subcohort (strata), age (timescale), sex (strata), and year of baseline visit, marital status, body-mass index, smoking (status, duration, intensity, intensity squared), employment status, education, and area-level socioeconomic status (2001 mean income on a neighborhood level).

EPIC-NL: HRs adjusted for subcohort (strata), age (timescale), sex (strata), and year of baseline visit, marital status, body-mass index, smoking (status, duration, intensity, intensity squared), employment status, education, and area-level socioeconomic status (2001 mean income on a neighborhood level).

KORA: HRs adjusted for subcohort (strata), age (timescale), sex (strata), and year of baseline visit, marital status, body-mass index, smoking (status, duration, intensity, intensity squared), employment status, education, and area-level socioeconomic status (Percentage of households with low income per 5 km² grid cell in 2007).

**Appendix VI:** **Synergistic effects of NDVI and air pollution; warm season temperature, cohort-specific and pooled results**

**Figures S23 to S34.** Forest plots of cohort-specific and pooled HRs for the interactions of PM_2.5_, NO_2_ and mean temperature warm season with NDVI at 10^th^ and 90^th^ percentile Hazard Ratios (HR) and 95% Confidence Intervals (95% CI) from Cox main model 3.

***S23.***

***
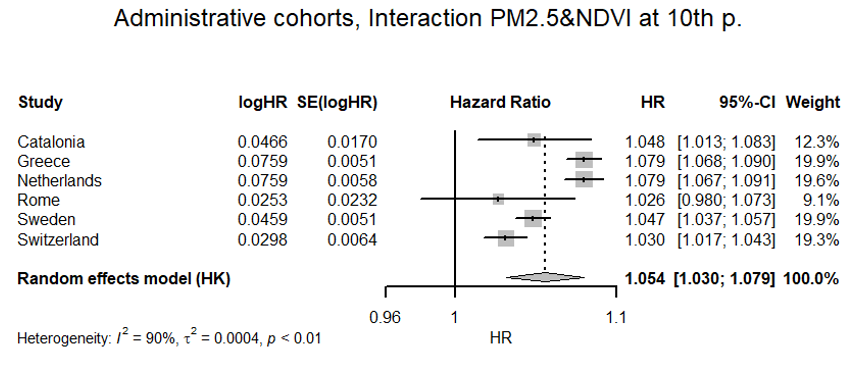
***

***S24.***

***
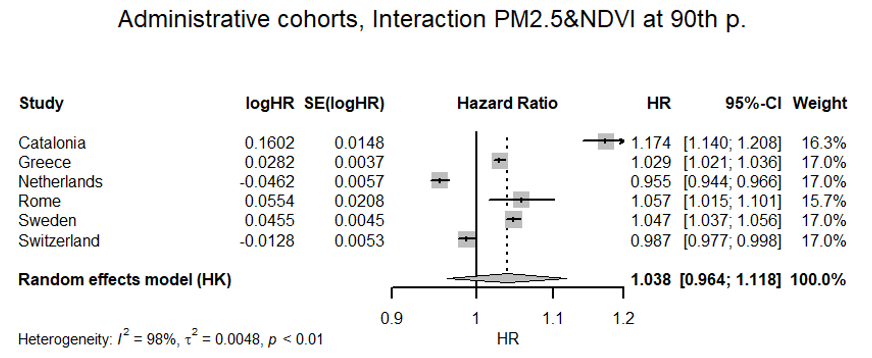
***

***S25.***

***
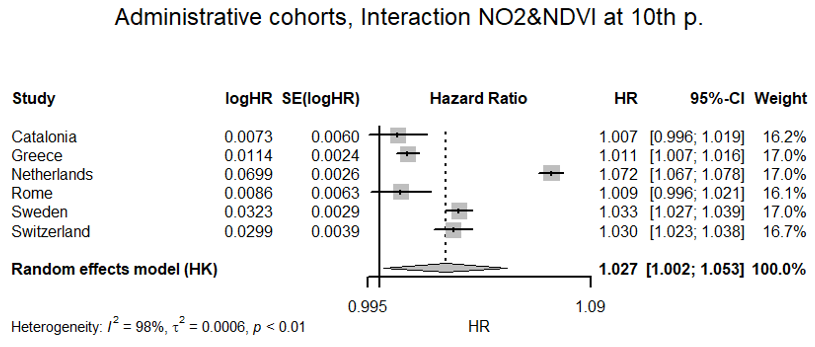
***

***S26.***

***
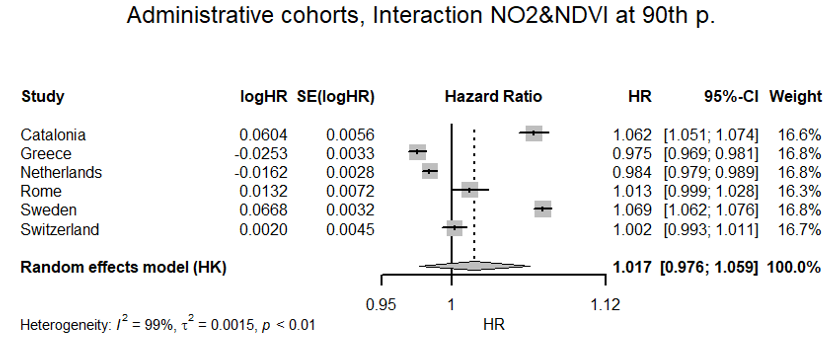
***

***S27.***

***
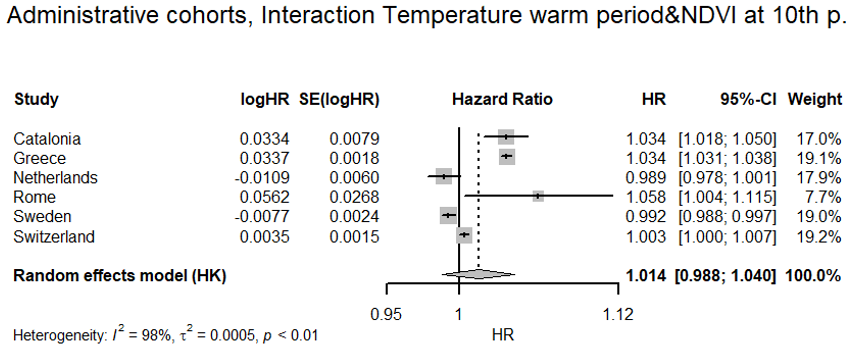
***

***S28.***

***
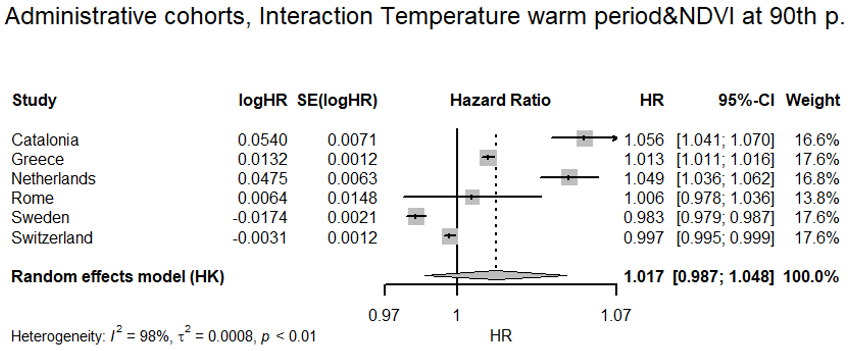
***

***S29.***

***
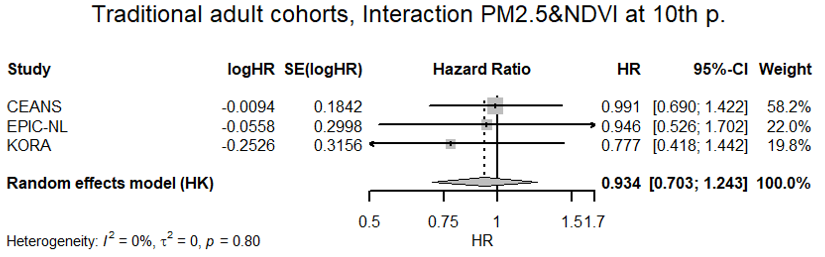
***

***S30.***

***
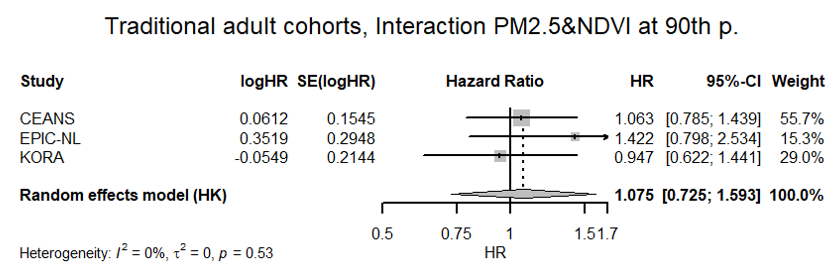
***

***S31.***

***
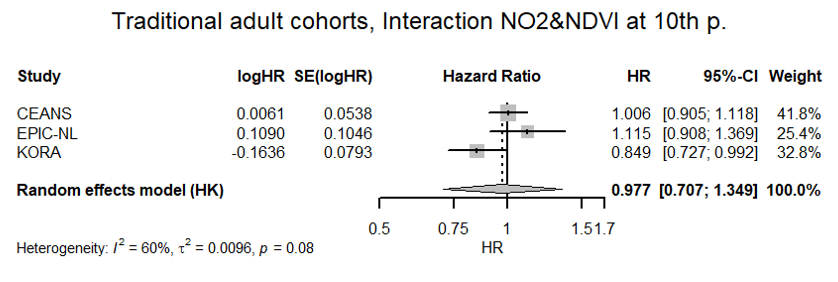
***

***S32.***

***
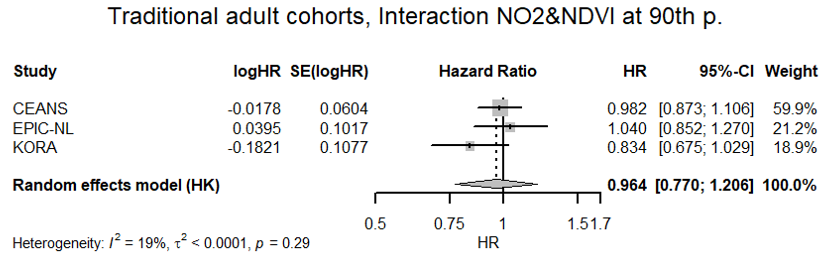
***

***S33.***

***
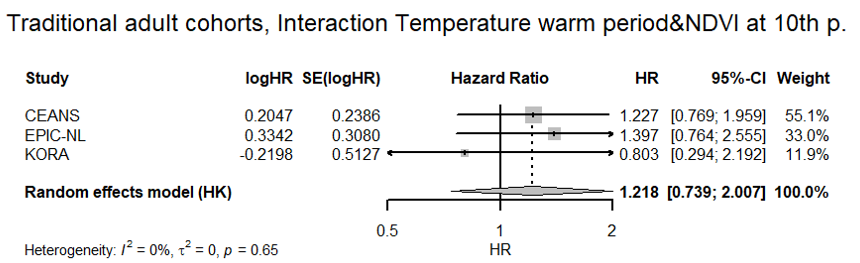
***

***S34.***

***
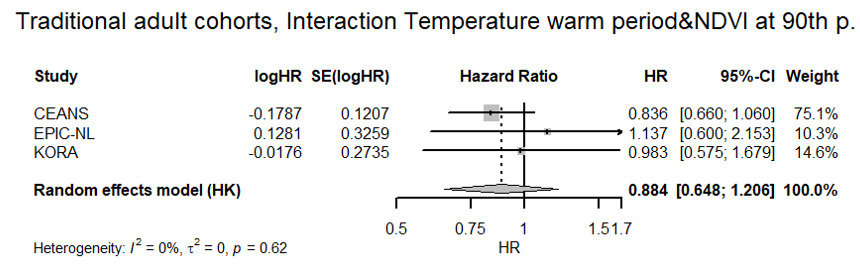
***

HRs are expressed per fixed increment.

Catalonia: HRs adjusted for age (time scale), sex (strata), smoking status, individual income, psca index, percentage of non-Spanish residents in census tract, and population density per m2.

Greece: HRs adjusted for age (time scale), sex (strata), NUTS1 areas country-wide (4 levels: Attica / Aegean Islands, Crete / North Greece / Central Greece)) & 4 area-level variables: tertiary education rate, unemployment rate, degree of urbanicity in 3 categories: 1. Cities (densely populated areas), 2. Towns and Suburbs (intermediate density areas) and 3. Rural areas (thinly populated areas) and married rate. For the Greater Area of Athens and other large municipalities (population greater than 100,000 inhabitants) in Greece, the aforementioned variables were available at square-block level. For the rest of the areas in Greece, the variables were available at municipality unit level.

Rome: HRs adjusted for age (timescale), sex (strata), place of birth, education level, employment status, marital status, citizenship, deprivation index on a census block level and unemployment rate, percentage of graduates and house prices on a neighborhood level.

Sweden: HRs adjusted for age (time scale), sex (strata), living condition, education level, district mean income, portion of people with high school or higher education in district, area.

Switzerland: HRs adjusted for age (time scale), strata(sex), Swiss region (n=7), marital status, occupational status, origin (i.e. Swiss vs. other), language region, socio-economic position index (SEP), community-level SEP index and community-level unemployment rate.

The Netherlands: HRs adjusted for age (time scale), sex (strata), area, wealth at 2010, categorized in deciles, partner status at 2010, individual socioeconomical status, area-level socio-economic status, area-level mean income at 2010, percentage of low-income households, urbanicity.

CEANS: HRs adjusted for subcohort (strata), age (timescale), sex (strata), and year of baseline visit, marital status, body-mass index, smoking (status, duration, intensity, intensity squared), employment status, education, and area-level socioeconomic status (2001 mean income on a neighborhood level).

EPIC-NL: HRs adjusted for subcohort (strata), age (timescale), sex (strata), and year of baseline visit, marital status, body-mass index, smoking (status, duration, intensity, intensity squared), employment status, education, and area-level socioeconomic status (2001 mean income on a neighborhood level).

KORA: HRs adjusted for subcohort (strata), age (timescale), sex (strata), and year of baseline visit, marital status, body-mass index, smoking (status, duration, intensity, intensity squared), employment status, education, and area-level socioeconomic status (Percentage of households with low income per 5 km² grid cell in 2007).

**Appendix VII:** **Multiple exposure models (across domains) & Cumulative Risk Index**

**Figures S35. to S46.** Cohort-specific and pooled Hazard ratios (HRs) with corresponding 95% Confidence Intervals (CIs) for the association of all-cause mortality with PM_2.5_ and NO_2_ from a) further adjusting for NDVI and mean warm temperature and b) the cumulative effect of the three exposures from a). All results are based on main model 3.

***S35.***

***
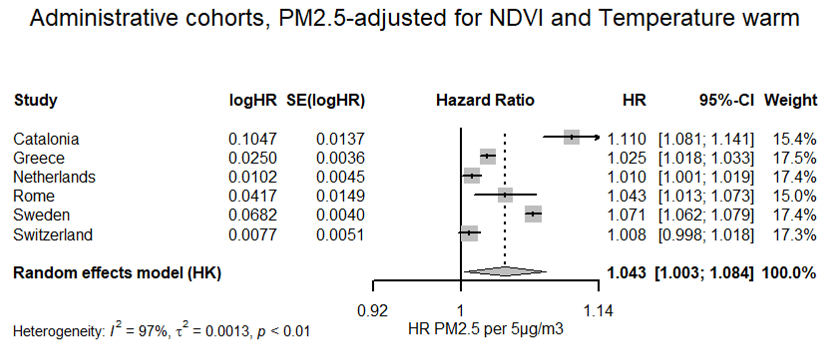
***

***S36.***

***
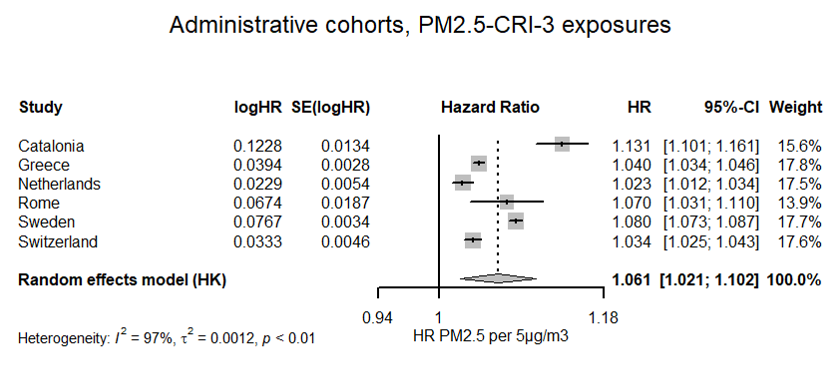
***

***S37.***

***
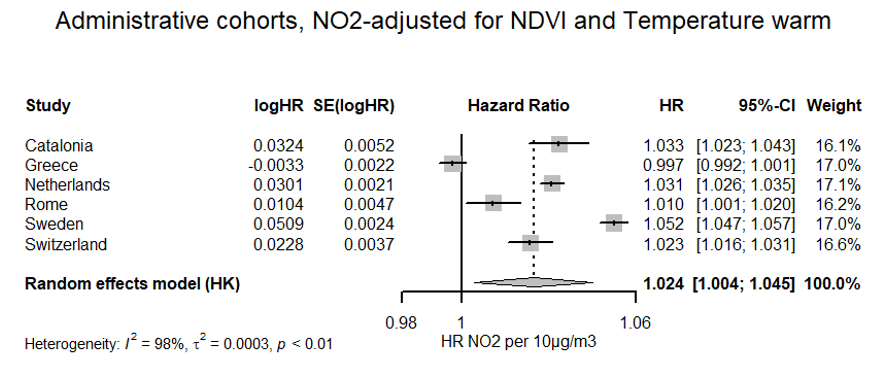
***

***S38.***

***
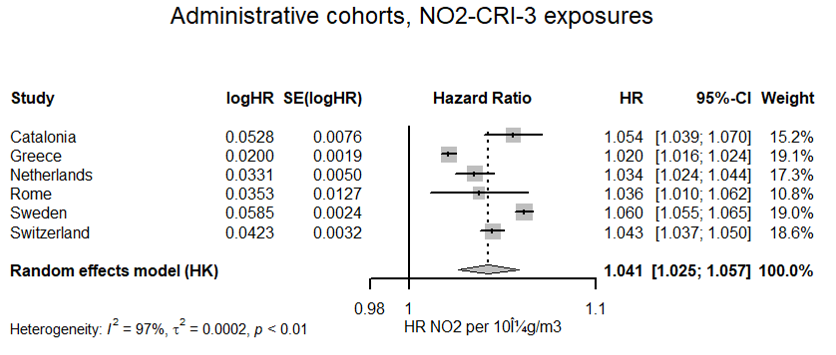
***

***S39.***

***
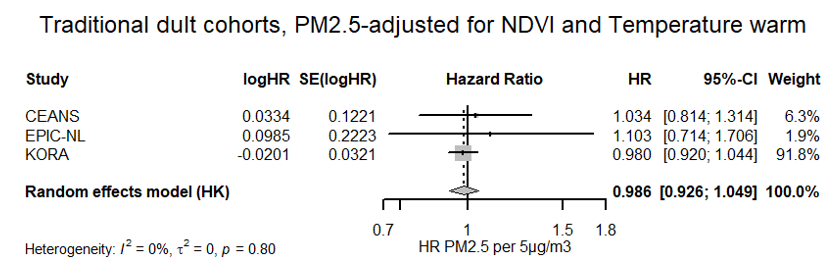
***

***S40.***

***
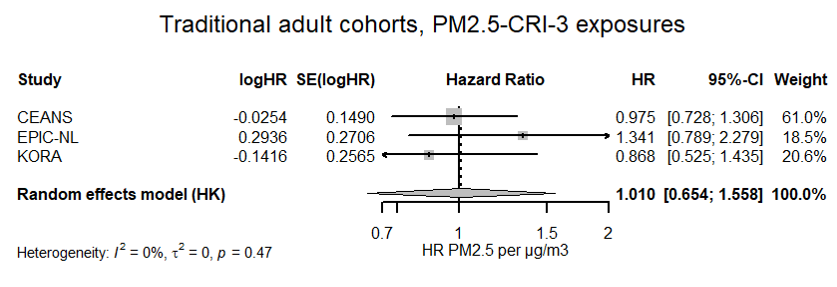
***

***S41.***

***
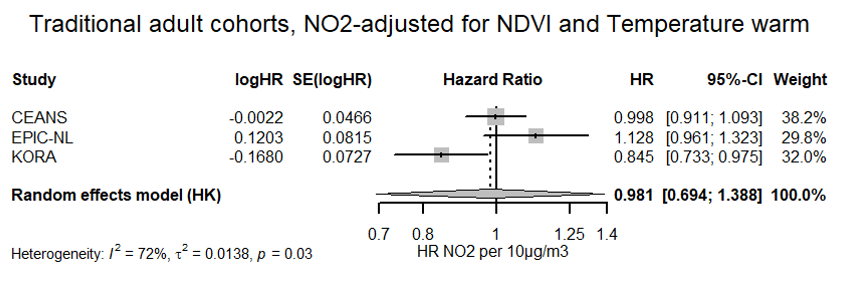
***

***S42.***

***
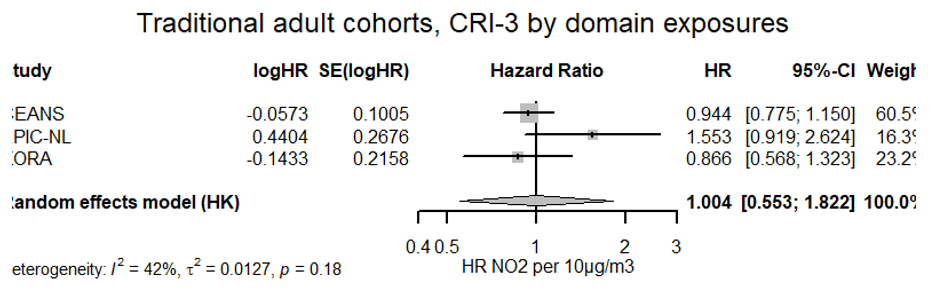
***

HRs are expressed per fixed increment. NDVI is expressed per 0.1 unit decrease.

Catalonia: HRs adjusted for age (time scale), sex (strata), smoking status, individual income, psca index, percentage of non-Spanish residents in census tract, and population density per m2.

Greece: HRs adjusted for age (time scale), sex (strata), NUTS1 areas country-wide (4 levels: Attica / Aegean Islands, Crete / North Greece / Central Greece)) & 4 area-level variables: tertiary education rate, unemployment rate, degree of urbanicity in 3 categories: 1. Cities (densely populated areas), 2. Towns and Suburbs (intermediate density areas) and 3. Rural areas (thinly populated areas) and married rate. For the Greater Area of Athens and other large municipalities (population greater than 100,000 inhabitants) in Greece, the aforementioned variables were available at square-block level. For the rest of the areas in Greece, the variables were available at municipality unit level.

Rome: HRs adjusted for age (timescale), sex (strata), place of birth, education level, employment status, marital status, citizenship, deprivation index on a census block level and unemployment rate, percentage of graduates and house prices on a neighborhood level.

Sweden: HRs adjusted for age (time scale), sex (strata), living condition, education level, district mean income, portion of people with high school or higher education in district, area.

Switzerland: HRs adjusted for age (time scale), strata(sex), Swiss region (n=7), marital status, occupational status, origin (i.e. Swiss vs. other), language region, socio-economic position index (SEP), community-level SEP index and community-level unemployment rate.

The Netherlands: HRs adjusted for age (time scale), sex (strata), area, wealth at 2010, categorized in deciles, partner status at 2010, individual socioeconomical status, area-level socio-economic status, area-level mean income at 2010, percentage of low-income households, urbanicity.

CEANS: HRs adjusted for subcohort (strata), age (timescale), sex (strata), and year of baseline visit, marital status, body-mass index, smoking (status, duration, intensity, intensity squared), employment status, education, and area-level socioeconomic status (2001 mean income on a neighborhood level).

EPIC-NL: HRs adjusted for subcohort (strata), age (timescale), sex (strata), and year of baseline visit, marital status, body-mass index, smoking (status, duration, intensity, intensity squared), employment status, education, and area-level socioeconomic status (2001 mean income on a neighborhood level).

KORA: HRs adjusted for subcohort (strata), age (timescale), sex (strata), and year of baseline visit, marital status, body-mass index, smoking (status, duration, intensity, intensity squared), employment status, education, and area-level socioeconomic status (Percentage of households with low income per 5 km² grid cell in 2007).

**Appendix VIII:** **Multiple exposure models same-domain & Cumulative Risk Index**

**Figures S43. to S52.** Forest plots of cohort-specific and pooled cumulative effect HRs for the associations of all-cause mortality and multi-same domain exposures. Cohort-specific and pooled Hazard ratios (HRs) with corresponding 95% Confidence Intervals (CIs) based on main model 3.

***S43.***

***
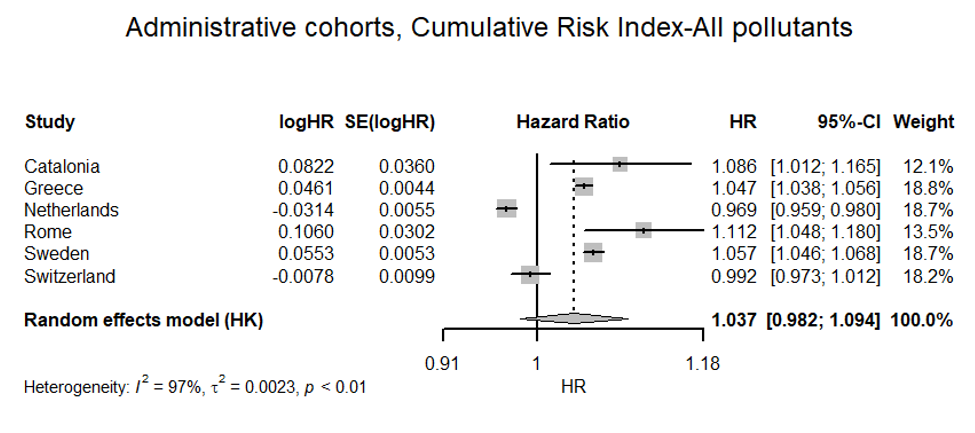
***

***S44.***

***
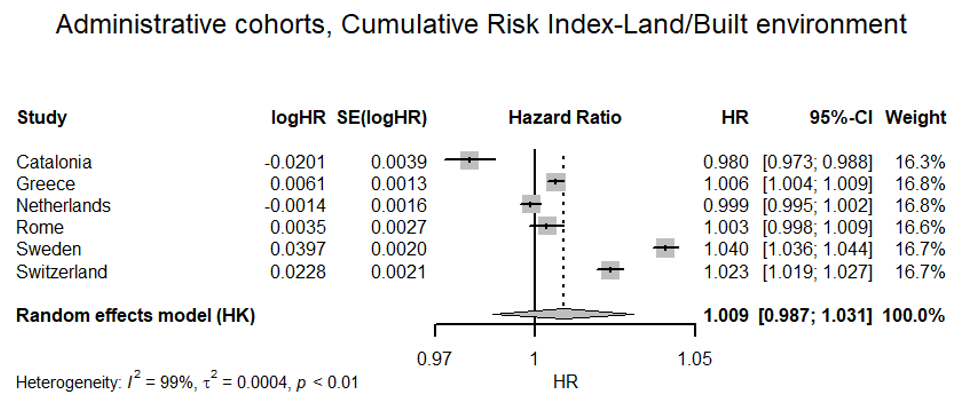
***

***S45.***

***
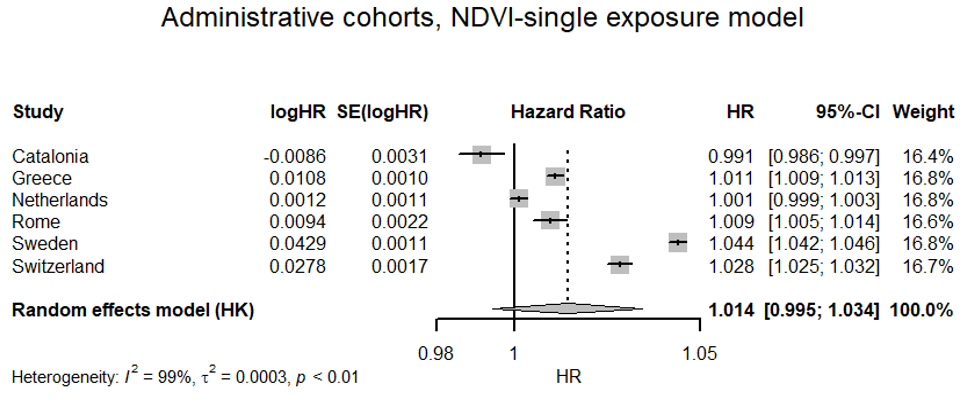
***

***S46.***

***
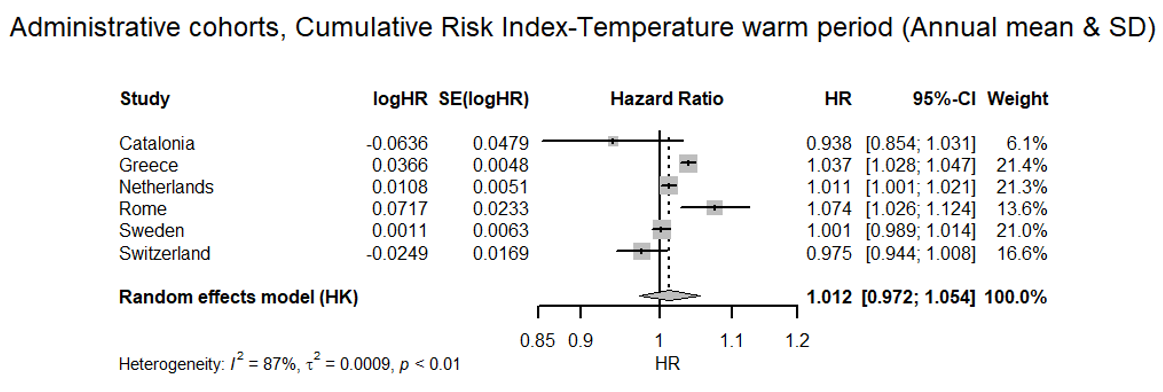
***

***S47.***

***
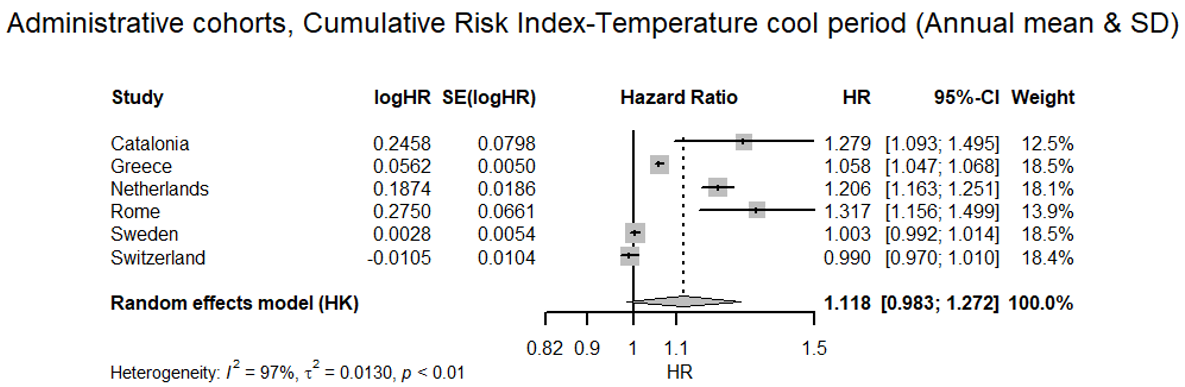
***

***S48.***

***
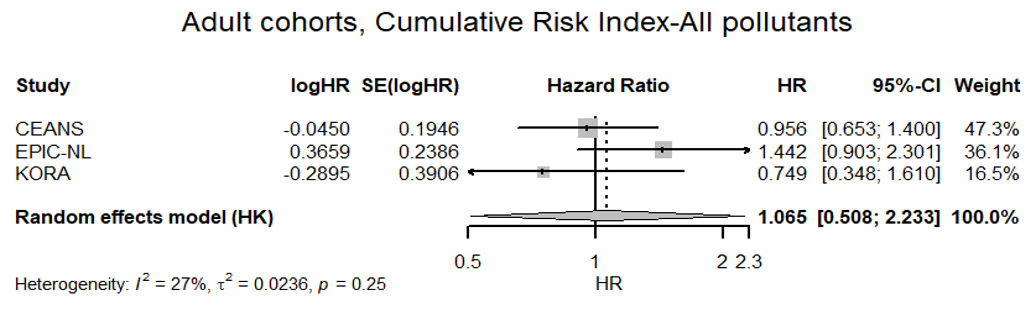
***

***S49.***

***
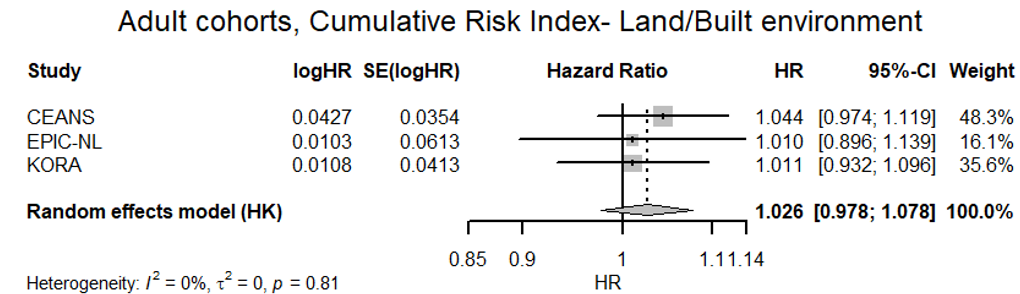
***

***S50.***

***
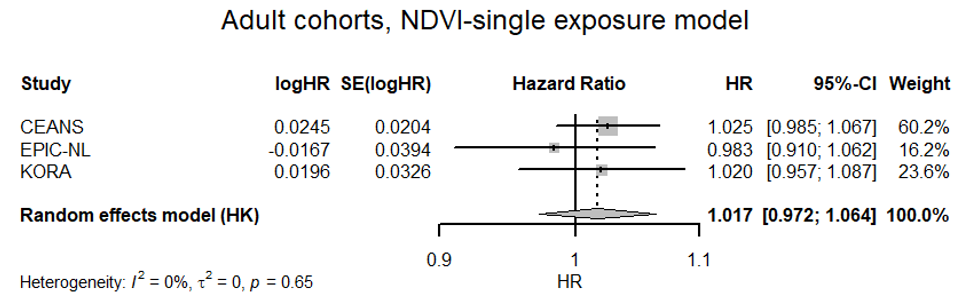
***

***S51.***

***
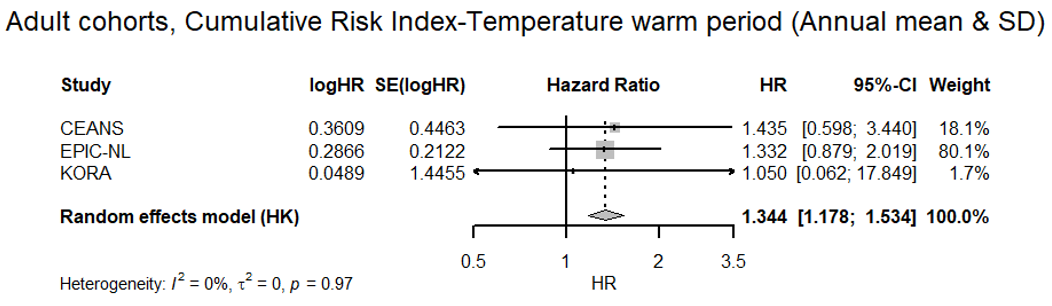
***

***S52.***

***
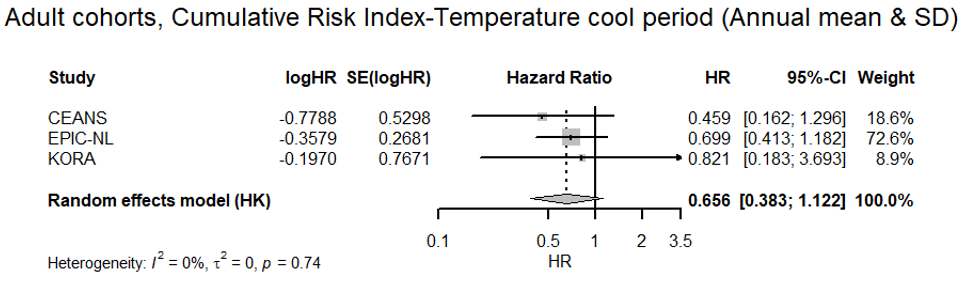
***

HRs are expressed per fixed increment. NDVI is expressed per 0.1 unit decrease.

Catalonia: HRs adjusted for age (time scale), sex (strata), smoking status, individual income, psca index, percentage of non-Spanish residents in census tract, and population density per m2.

Greece: HRs adjusted for age (time scale), sex (strata), NUTS1 areas country-wide (4 levels: Attica / Aegean Islands, Crete / North Greece / Central Greece)) & 4 area-level variables: tertiary education rate, unemployment rate, degree of urbanicity in 3 categories: 1. Cities (densely populated areas), 2. Towns and Suburbs (intermediate density areas) and 3. Rural areas (thinly populated areas) and married rate. For the Greater Area of Athens and other large municipalities (population greater than 100,000 inhabitants) in Greece, the aforementioned variables were available at square-block level. For the rest of the areas in Greece, the variables were available at municipality unit level.

Rome: HRs adjusted for age (timescale), sex (strata), place of birth, education level, employment status, marital status, citizenship, deprivation index on a census block level and unemployment rate, percentage of graduates and house prices on a neighborhood level.

Sweden: HRs adjusted for age (time scale), sex (strata), living condition, education level, district mean income, portion of people with high school or higher education in district, area.

Switzerland: HRs adjusted for age (time scale), strata(sex), Swiss region (n=7), marital status, occupational status, origin (i.e. Swiss vs. other), language region, socio-economic position index (SEP), community-level SEP index and community-level unemployment rate.

The Netherlands: HRs adjusted for age (time scale), sex (strata), area, wealth at 2010, categorized in deciles, partner status at 2010, individual socioeconomical status, area-level socio-economic status, area-level mean income at 2010, percentage of low-income households, urbanicity.

CEANS: HRs adjusted for subcohort (strata), age (timescale), sex (strata), and year of baseline visit, marital status, body-mass index, smoking (status, duration, intensity, intensity squared), employment status, education, and area-level socioeconomic status (2001 mean income on a neighborhood level).

EPIC-NL: HRs adjusted for subcohort (strata), age (timescale), sex (strata), and year of baseline visit, marital status, body-mass index, smoking (status, duration, intensity, intensity squared), employment status, education, and area-level socioeconomic status (2001 mean income on a neighborhood level).

KORA: HRs adjusted for subcohort (strata), age (timescale), sex (strata), and year of baseline visit, marital status, body-mass index, smoking (status, duration, intensity, intensity squared), employment status, education, and area-level socioeconomic status (Percentage of households with low income per 5 km² grid cell in 2007).
